# Far-Red Light Coordinates the Diurnal Changes in the Transcripts Related to Nitrate Reduction, Glutathione Metabolism and Antioxidant Enzymes in Barley

**DOI:** 10.3390/ijms23137479

**Published:** 2022-07-05

**Authors:** Eszter Balogh, Balázs Kalapos, Mohamed Ahres, Ákos Boldizsár, Krisztián Gierczik, Zsolt Gulyás, Mónika Gyugos, Gabriella Szalai, Aliz Novák, Gábor Kocsy

**Affiliations:** Agricultural Institute, Centre for Agricultural Research, 2462 Martonvásár, Hungary; balogheszt@gmail.com (E.B.); kalapos.balazs@atk.hu (B.K.); mohamed.ahres@atk.hu (M.A.); akos.boldizsar@gmail.com (Á.B.); gierczik.krisztian@atk.hu (K.G.); gulyas.zsolt@atk.hu (Z.G.); gyugos.monika@atk.hu (M.G.); szalai.gabriella@atk.hu (G.S.); novak.aliz@atk.hu (A.N.)

**Keywords:** antioxidants, circadian clock, light spectrum, nitrate reduction, redox-responsive transcription factors, sulfate reduction

## Abstract

Spectral quality, intensity and period of light modify many regulatory and stress signaling pathways in plants. Both nitrate and sulfate assimilations must be synchronized with photosynthesis, which ensures energy and reductants for these pathways. However, photosynthesis is also a source of reactive oxygen species, whose levels are controlled by glutathione and other antioxidants. In this study, we investigated the effect of supplemental far-red (735 nm) and blue (450 nm) lights on the diurnal expression of the genes related to photoreceptors, the circadian clock, nitrate reduction, glutathione metabolism and various antioxidants in barley. The maximum expression of the investigated four photoreceptor and three clock-associated genes during the light period was followed by the peaking of the transcripts of the three redox-responsive transcription factors during the dark phase, while most of the nitrate and sulfate reduction, glutathione metabolism and antioxidant-enzyme-related genes exhibited high expression during light exposure in plants grown in light/dark cycles for two days. These oscillations changed or disappeared in constant white light during the subsequent two days. Supplemental far-red light induced the activation of most of the studied genes, while supplemental blue light did not affect or inhibited them during light/dark cycles. However, in constant light, several genes exhibited greater expression in blue light than in white and far-red lights. Based on a correlation analysis of the gene expression data, we propose a major role of far-red light in the coordinated transcriptional adjustment of nitrate reduction, glutathione metabolism and antioxidant enzymes to changes of the light spectrum.

## 1. Introduction

Spectral quality, intensity and period of light modify many signaling and regulatory pathways, controlling the ability for stress adaptation [1,2,3,4]. The various photoreceptors sense the individual spectral components (phytochromes for red and far-red lights, cryptochromes for blue light), the amount and the ratio of which change during the day [5,6,7]. The proper integration of external signals with diurnal oscillations is maintained by the circadian clock, an endogenous oscillator mechanism that produces biological rhythms with approximately 24 h periods. Thus, it is able to synchronize developmental and metabolic processes with daily changes in the environmental factors, such as light and temperature [8,9,10]. In the model plant Arabidopsis, the circadian rhythm is defined by so called core oscillator genes, and comprises three interlocking feedback loops [11,12]. The “morning loop” involves CIRCADIAN CLOCK-ASSOCIATED 1 (CCA1) and LONG ELONGATED HYPOCOTYL (LHY) transcription factors coding genes that are co-expressed in the early morning [13,14]. During the afternoon and night, their expression is repressed by PSEUDO RESPONSE REGULATOR (PRR) and TIMING OF CAB EXPRESSION 1 (TOC1) proteins, and released again by the “evening complex” formed by EARLY FLOWERING 3 and 4 (ELF3, ELF4) and LUX ARRYTHMO (LUX) proteins [11,12,15,16]. Some Arabidopsis clock gene orthologs have been identified in rice (Oryza sativa) [16,17], barley (*Hordeum vulgare*) [10,18,19] and *Lemna* species [20,21]. In barley, a CCA1 orthologue and also five distinctive PRR sequences were identified: PRR1/TOC1, PPR73, PRR37, PRR95 and PRR59 [10]. The photo-responsive PRR37 (Ppd-H1) is responsible for photoperiod adaptation, which is orthologous to the AtPRR7, and its natural recessive mutation causes photoperiod insensitivity and late flowering in spring barley genotypes [18,19]. These results suggest that circadian clock genes are highly conserved, and their cycling patterns also show high similarities across monocot and dicot species.

A global analysis revealed that approximately 30% of the Arabidopsis transcriptome is clock-regulated under day/night growing conditions [9]. In maize (*Zea mays*), over 13,000 transcripts were examined, and approximately 10% displayed circadian expression patterns [22]. According to a comparison of different microarray databases, photocycles and thermocycles regulated approximately 60% of the expressed nuclear genes in rice (*Oryza sativa*) and poplar (*Populus trichocarpa*) [23]. Several genes showing circadian or diurnal rhythm encode enzymes of metabolic pathways related to the fine regulation of the cellular redox state, which is either genetically determined or triggered by environmental changes [24,25]. Plants developed enzymatic and non-enzymatic scavenging systems in order to prevent the overproduction of reactive oxygen species (ROS), causing oxidative stress due to the unbalanced cellular redox state [24,26]. The expression profiles of several antioxidant enzymes contributing to the elimination of ROS molecules, such as ascorbate peroxidases (APX), catalases (CAT) and peroxiredoxins (PRX), were reported to change according to a diurnal rhythm [27,28,29,30,31,32,33].

A central component of the antioxidant system is glutathione (GSH), a low-molecular-weight thiol [24]. It is used both for the transmission of redox signals and, as part of the ascorbate–glutathione cycle, for detoxification of ROS. Due to its central cysteine, GSH can be easily oxidized to glutathione disulphide (GSSG) during the scavenging process and then converted back by glutathione reductase (GR), maintaining high levels of its reduced form under stress conditions [25,34,35,36]. Some members of the GSH biosynthetic pathway are also light-inducible, such as adenosine-5′-phosphosulfate-reductase (APSR), whose activity is characterized by diurnal rhythm [37,38]. Cysteine, a key metabolite of GSH production, is the end product of sulfate assimilation and it is required for various pathways in plants [39,40]. The nitrogen is provided for cysteine formation through *O*-acetyl-l-serine, which connects sulfate and nitrate reductions. Similarly to sulfate reduction, nitrate reduction also exhibits a diurnal rhythm [41,42].

Both light intensity and spectrum have an impact on the plant cellular redox state and the corresponding metabolic pathways. Photoreceptor molecules, such as phytochromes and cryptochromes, are involved in the control of the various plant developmental processes (e.g., circadian clock and photoperiod-dependent flowering) and biotic and abiotic stress responses [2,3,43,44,45,46,47,48]. There are many reports about the stimulating effect of various red/far-red and blue light LED treatments on the antioxidant enzyme system, especially in the case of in vitro plant morphogenesis and acclimation [49]. ROS-scavenging enzymes, such as CAT and GST, were reported to be induced by red-light treatment in *Eleutherococcus senticosus* somatic embryos [50], and higher APX activities were observed under fluorescent white and blue lights than in red or far-red lights in induced adventitious roots of *Morinda citrifolia* [51]. A recent study revealed that the R/FR light ratio caused decrease in reduced ascorbate and GSH content, while interestingly, the ratio of oxidized forms (dehydroascorbate and GSSG) remained the same under both high- and low-R/RF conditions. The observed changes in ascorbate contents were achieved over a relatively short period of time, suggesting that the R/FR ratio is a key determinant of the extent of ascorbate accumulation and that the regulatory effect of R/FR ratio is triggered early in the acclimatization response to the changing light conditions [52]. In cyanobacterium *Synechocystis* sp., red light led to a 30% increase, while blue light and exposure to dark led to a 20–25% decline in GSH levels [53].

The spectral control of the diurnal expression rhythm of genes related to nitrate reduction, glutathione metabolism and antioxidant enzymes was not investigated in a previous complex study, and the results are expected contribute to the better understanding of this process. According to our hypothesis, blue and far-red lights may modify and coordinate the redox-dependent diurnal fluctuations in the transcript level of these genes. To test this assumption, the effect of supplemental far-red and blue lights on the diurnal expression of the genes related to red/far-red and blue light photoreceptors, the circadian clock, redox control, nitrate reduction, glutathione metabolism and various antioxidant enzymes was investigated in barley.

## 2. Results

### 2.1. Effect of Light Spectrum on Photoreceptor-Coding Genes

**Figure 1:**
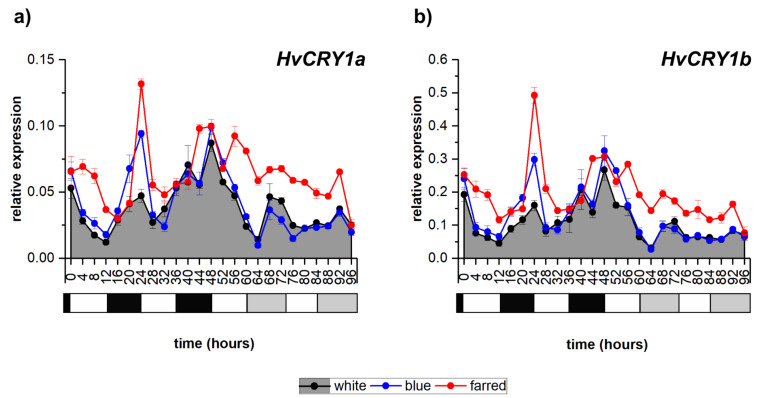
Expression patterns of barley cryptochrome-coding genes *HvCRY1a* (**a**) and *HvCRY1b* (**b**) under white, blue and far-red illumination. Values on the X axes show the time in hours after the start of the experiment; white and black bars below them indicate the light and dark periods, while gray bars indicate the subjective “night” period during constant-light conditions. Transcript levels were calculated with ΔCt method, with expression data and error bars indicating standard deviations were calculated using three biological replicates. The following significant differences were calculated at *p* ≤ 5% level: *HvCRY1a*—0.015; *HvCRY1b*—0.028.

Additional far-red (FR) illumination greatly modified the amplitude and periodicity of transcript levels of all four investigated barley photoreceptor coding genes compared to the normal white (W) light condition, while blue (B) had only smaller effects on their expression (Figure 1a,b and Supplementary Figure S2a,b). The expression of *HvPHYA* shows a clear circadian pathway under additional FR light, while it changed to diurnal under B-light conditions (Supplementary Figure S2a). Cryptochrome-coding genes (*HvCRY1a*, *HvCRY1b* and *HvCRY2*) also presented diurnal expression patterns under all light conditions (except for *HvCRY2* under W light). Additional FR illumination raised the expression level of cryptochrome-coding genes, while interestingly additional B light did not have a similar effect. The expression level of all photoreceptor-coding genes peaked during the dark period, except for *HvCRY2* under supplemental FR illumination.

### 2.2. Expression of Circadian Clock Genes

**Figure 2:**
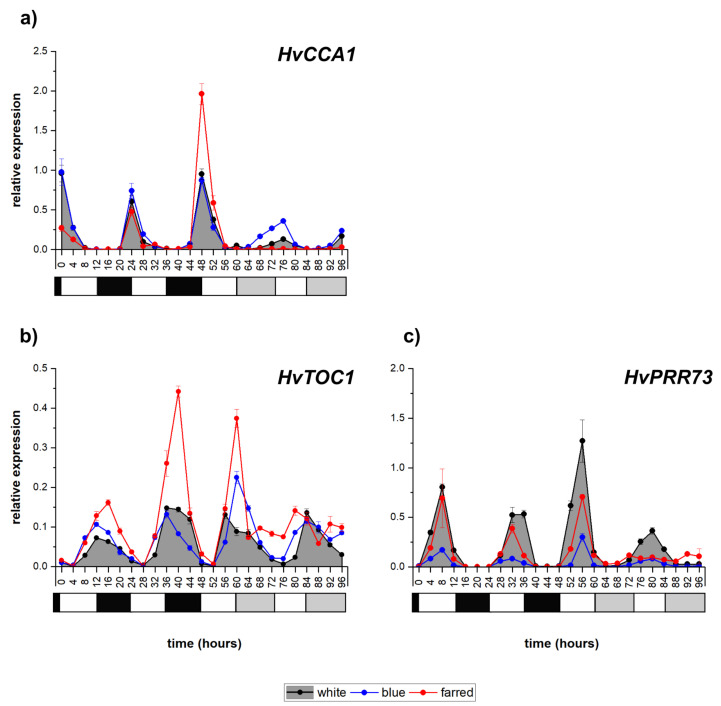
Expression patterns of barley circadian clock genes *HvCCA1* (**a**), *HvTOC* (**b**) and *HvPRR73* (**c**) with white, blue and far-red illumination. Calculating methods and the legends of graphs are the same as described in Figure 1. The following significant differences were calculated at *p* ≤ 5% level: *HvCCA1*—0.16; *HvTOC*—0.023; *HvPRR73*—0.059.

The expression analysis revealed that the transcription of *HvCCA1* (Figure 2a) is highly induced at dawn, and it shows a circadian pattern in W and additional B light, although with modest oscillations under constant illumination. However, under supplemental FR light resulting in a low R/FR ratio, the rhythmicity was diurnal since it was not detectable under constant light. The expression of *HvTOC1* peaked early in the dark period and kept its periodicity and amplitudes during constant light conditions, showing circadian rhythm under W and additional B illumination (Figure 2b). Additional FR illumination raised the level of *HvTOC1* expression under normal light/dark cycles; however, under constant light, the amplitude was severely reduced. In normal W-light illumination, the transcript level of *HvPRR73* peaked halfway through the light period and kept its periodicity under constant-light conditions, showing the circadian rhythm (Figure 2c). Additional FR and especially B illumination reduced the amplitude of *HvPRR73* expression, and the periodicity disappeared under constant-light conditions.

### 2.3. Analysis of Transcription Factors Related to Redox Regulation

**Figure 3:**
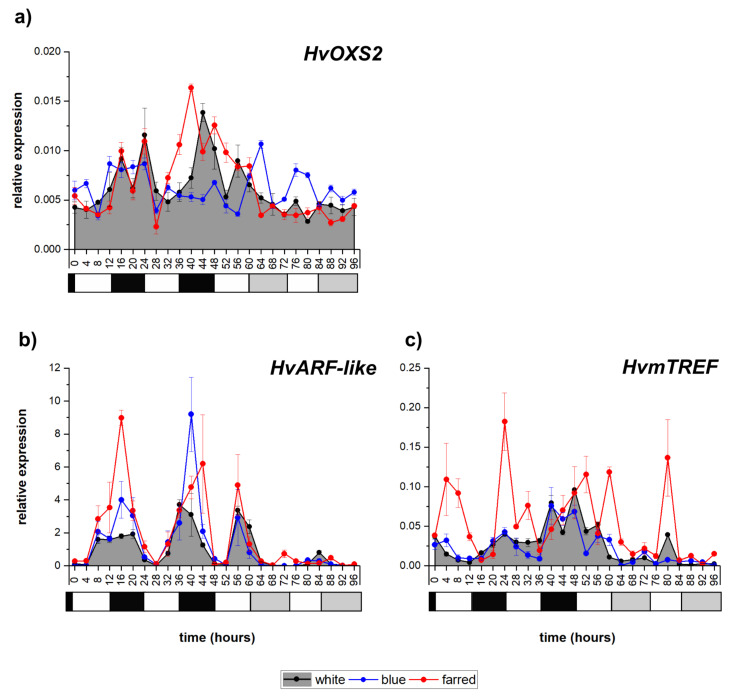
Expression patterns of *HvOXS2* (**a**), *HvARF-like* (**b**) and *HvmTREF* (**c**) transcription factor coding genes under white, blue and far-red light illumination. Calculating methods and the legends of graphs are the same as described in Figure 1. The following significant differences were calculated at *p* ≤ 5% level: *HvOXS2*- 0.00059; *HvARF-like*—0.37; *HvmTREF*—0.0068.

From the investigated redox-responsive transcription factors, the expression of a *H. vulgare* auxin-response-factor-coding gene (*HvARF-like)* showed a clear diurnal pattern with peaking during dusk, while the number of oxidative stress 2 and mitochondrial transcription termination factor (*HvOXS2* and *HvmTREF)* transcripts gradually increased during the dark in light/dark cycles under W illumination with smaller oscillations (Figure 3). Their oscillations became very low or disappeared in constant white light. The expression of *HvOXS2* was greatly increased by additional B light under constant illumination, while that of *HvARF-like* was greatly induced both by FR and by B in light/dark cycles. The peaks of the *HvmTREF* transcript exhibited a great increase in FR light and could be observed in both cycling and constant light.

### 2.4. Influence of Light Spectrum on Genes Related to Nitrate Reduction

**Figure 4:**
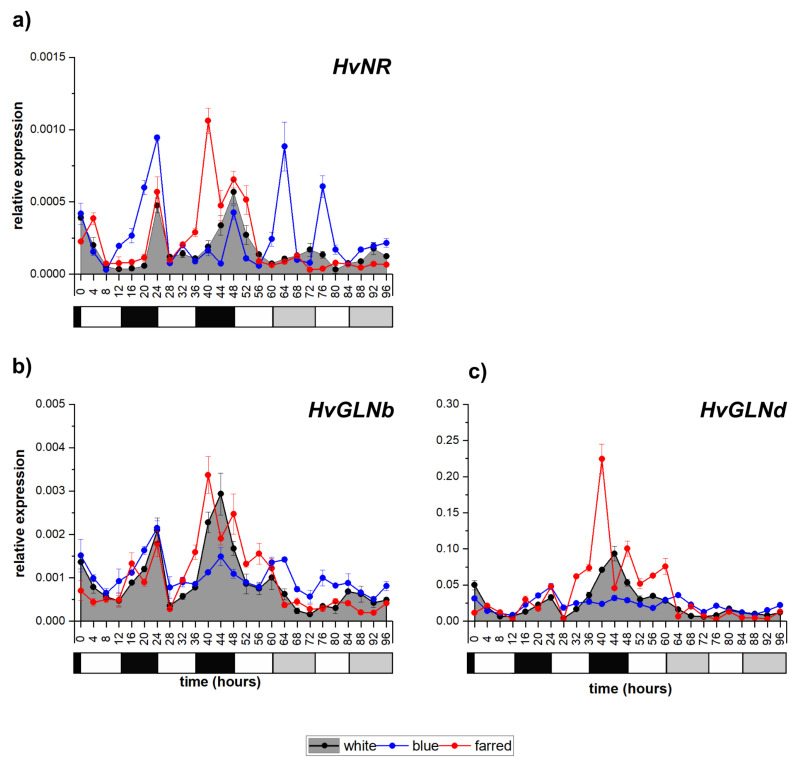
Expression patterns of *H. vulgare* genes related to nitrate reduction: nitrate reductase (*HvNR)* (**a**) glutamine synthetase-b (*HvGLNb*) (**b**) and glutamine synthetase-d (*HvGLNd*) (**c**) white, blue and far-red light illumination, respectively. Calculating methods and the legends of graphs are the same as described in Figure 1. The following significant differences were calculated at *p* ≤ 5% level: *HvNR*—0.00012, *HvGLNb—*0.00035; *HvGLNd*—0.0078.

Concerning the investigated nitrate-reduction-related genes, the transcription of nitrate reductase (*HvNR*) had a circadian pattern in W light, peaking at the end of the dark period (Figure 4a). Under constant-light conditions, the amplitude of gene expression severely decreased in W and additional FR illumination. However, supplemental B light increased the level of expression, and an extra peak appeared during constant illumination as well.

In the case of the barley glutamine-synthetase-coding genes, only *HvGLNb* and *HvGLNd* presented a diurnal expression pattern in normal W illumination, which disappeared under constant light (Figure 4b,c). *HvGLNa* also had some changes in its transcript levels, but it was not associated with the day/night shift (Supplementary Figure S2c), and the expression of *HvGLNe* was barely detectable (Supplementary Figure S2d). Additional FR illumination raised the expression level of *HvGLNa* and *HvGLNd* but did not cause periodicity. Supplemental B light increased the amplitude and modified the expression pattern of *HvGLNa* and *HvGLNe* in an irregular manner, but reduced the transcript level in the case of *HvGLNb* and *HvGLNd* during the 2nd day.

### 2.5. Diurnal Oscillations in Transcripts Related to Glutathione Metabolism

**Figure 5:**
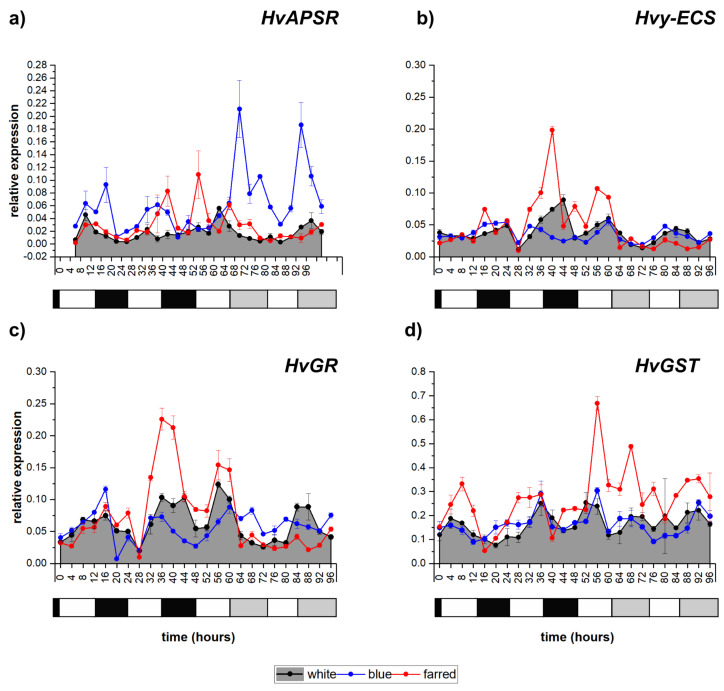
Expression patterns of *H. vulgare* genes related to glutathione metabolism: APS reductase (*HvAPSR)*, (**a**) γ-glutamylcysteine synthetase1 (*Hv*γ-*ECS*), (**b**) glutathione reductase (*HvGR)* (**c**) and glutathione S-transferase (*HvGST)* (**d**), under white, blue and far-red light illumination, respectively. Calculating methods and the legends of graphs are the same as described in Figure 1. The following significant differences were calculated at *p* ≤ 5% level: *HvAPSR—*0.019; *Hv*γ-*ECS*—0.023; *HvGR—*0.016; *HvGST*—0.019.

*H. vulgare* APS reductase (*HvAPSR*) was expressed at a low level, and the transcript peaked in the middle of the day, shifting to the middle of the “subjective dark” on the second day of constant W-light illumination (Figure 5a). Additional B and FR light increased the level of expression and enhanced the amplitude. Interestingly, in response to B illumination, a quasi-circadian pattern could be detected with maximums during the day in cycling light, and peaks were significantly higher and shifted to the “subjective dark” period under constant-light conditions. *Hvγ-ECS* also presented a low expression level and weak periodicity during the whole experiment in response to W and additional B light (Figure 5b). In white light, it peaked during dawn under light/dark cycles. FR illumination strongly enhanced its transcript level during the second dark period. The transcription of *HvGR* and *HvGST* showed how rhythmicity was connected to day/night shifts with an increase during the light and a decrease during the dark period, and the oscillations became lower in constant light (Figure 5c,d). Additional FR illumination enhanced the expression level of both genes during light/dark cycles; however, under constant light this effect persisted only in the case of *HvGST.*

### 2.6. Expression of Antioxidant-Enzyme-Coding Genes

**Figure 6:**
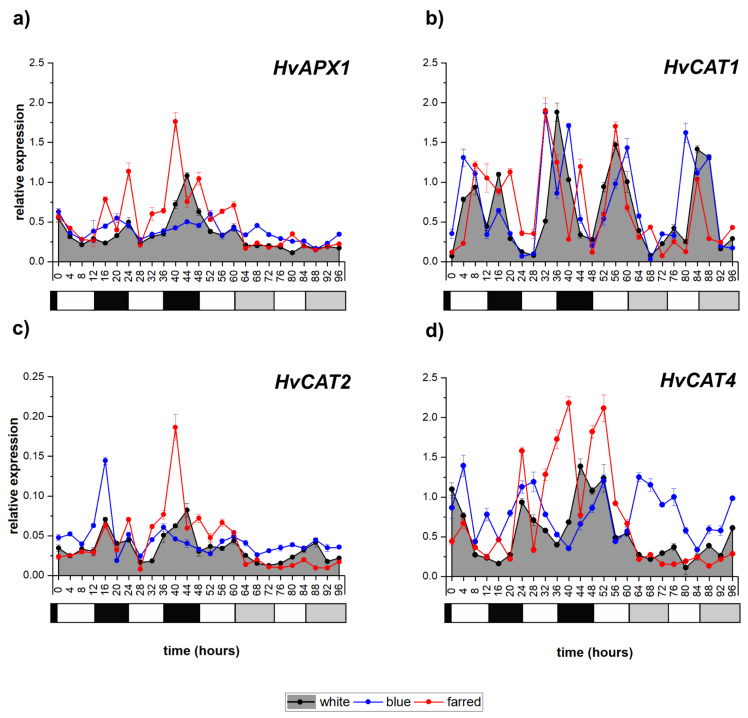
Expression patterns of *H. vulgare* antioxidant-enzyme-coding genes: ascorbate peroxidase 1 (*HvAPX1*) (**a**) and catalase (*HvCAT1-4)* (**b**–**d**), under white, blue and far-red light illumination, respectively. Calculating methods and the legends of graphs are the same as described in Figure 1. The following significant differences were calculated at *p* ≤ 5% level: *HvAPX1—*0.049; *HvCAT1—*0.17; *HvCAT2—*0.013; *HvCAT4—*0.21.

From the investigated ascorbate-peroxidase-coding genes, the expression of *HvAPX1* followed a diurnal rhythm during light/dark cycles under all conditions, but the peak during the second night was far greater under FR and smaller under B, respectively, compared to W light (Figure 6a). Oscillations in the transcript levels were lower for *HvAPX4* in the case of W light and even disappeared under B illumination (Supplementary Figure S2e). Under constant light, very low or zero fluctuation was observed in the case of both *HvAPX* genes.

Among catalase-coding genes, the expression of *HvCAT1* showed a clear circadian pattern under all light conditions, reaching maximum levels during the light period (Figure 6b). In contrast, *HvCAT2* and *4* exhibited their maximum expression level during the second part of the dark phase under cycling W light, and the peaks were shifted and became smaller in constant W light (Figure 6c,d). Supplemental B and FR lights increased their expression level (B light on the first day, FR on the second day of the experiment, respectively). Applying continuous illumination, transcript levels were higher under B light than under W and additional FR light. Under cycling W light, the transcription of peroxiredoxin (*HvPRX*) increased during the light period and decreased at the end of the dark phase, while oscillation disappeared in constant illumination (Supplementary Figure S2f). A similar pattern was detected under FR light while under B light constant transcript levels were observed under light/dark cycles and high amplitude during constant illumination.

### 2.7. Effect of Spectrum on the Amount and Redox State of Thiols

Changes in thiol content did not show any clear periodicity connected to day/night changes in light (Supplementary Figure S3). There was a moderate increase in the Cys and GSH contents during the second half of the light period in cycling white light, and they decreased in response to constant W light (Supplementary Figure S3a,b). Supplemental FR illumination was enhanced (except for Cys in cycling illumination), and B illumination lowered the level of reduced thiols (except in constant light). Additional B light caused a brief but large increase in oxidized thiol content during the first 24 h under constant-light conditions while FR light had a similar effect on the subsequent 24 h (Supplementary Figure S3c,d). Reduction potential of CyS/CySS and GSH/GSSG redox couples did not show any periodicity either (Supplementary Figure S3e,f). The highest reduction potential values were detected under cycling illumination with additional B light, and the lowest ones under constant illumination with additional FR light.

## 3. Discussion

Spectral composition and intensity of light fluctuate in spatial (latitude, altitude) and in temporal (daily, seasonal) manners as well [7]; thus, it is essential to synchronize metabolic processes with environmental signals to ensure proper development. The importance of the diurnal adjustment of gene expression in the adaptation to the changing light conditions was shown in barley, where an increase in the expression of a frost-tolerance-related C-repeat-binding-factor-coding gene was induced by a low R/FR ratio [3]. Such light conditions occur during sunset, and they allow the protection against the subsequent decrease in the temperature during the night. Similarly, a spectrum-dependent modification of the diurnal changes of the transcripts related to the nitrate reduction, glutathione metabolism and redox system was observed in the present study (Table 1), which makes possible the efficient adaptation of barley to the various light conditions during its growth in different cultivation areas.

In plants, both the phytochrome and cryptochrome types of photoreceptors are used for gathering information about the light environment to set the circadian clock [5,6,54,55]. *Arabidopsis* phyA and phyB act additively in R-light input to the clock, whereas cry1 and cry2 act redundantly in B-light input, and cry1 is also necessary for phyA signaling to the clock in both red and blue light spectra [54]. Additional FR light increased the expression of all evaluated cryptochrome- and phytochrome-coding genes in barley, and modified the amplitude and periodicity of *HvCRY2* and *HvPHYA* under constant-light conditions, suggesting a specific regulatory effect. The circadian clock genes are all probable targets of light signaling; however, the details of these pathways are not completely understood. The transduction of the light signal from phytochromes to core clock genes may involve several members of the *PIF3* family [56].

The circadian clock in plants consists of three regulatory loops: the morning and evening feedback loops and an interlocking central loop [11,12]. As was expected, the expression of the morning-loop-related *HvCCA1* was the highest at the beginning of the light period, while the activity of the evening-loop-related genes peaked at the end of the light period (*HvPRR73*) and at beginning of the dark period (*HvTOC1*), respectively. Interestingly, the expression of *HvCCA1* was barely detectable under constant-W-light conditions, while *HvTOC1* and *HvPRR73* showed periodic expression, although at a reduced level. In previous studies [3,57], barley circadian clock genes showed reduced, constant expression in continuous light or dark conditions; only *HvCCA1* showed gradually increasing expression in seedlings growing in constant darkness. Additional FR illumination led to the great reduction in or disappearance of the diurnal oscillations in the case of all the three genes, while additional B light resulted in reduced magnitude for *HvCCA1* and *HvPRR73* expression. This indicates the special regulatory effect of the individual spectral components. A similar effect of low R/FR on *HvCCA1* and *HvTOC1* was also shown in a previous study [3]. These results demonstrate that the W-light-induced entrainment can be preserved after the disappearance of the external rhythmicity only if the spectral condition remains constant. The importance of the spectral effect in this process was also revealed in *Arabidopsis*, where *AtCCA1* mRNA had a relatively long half-life in FR light but was not stable in red or B light [58].

The maximum transcript levels of the selected redox-related transcription factors [59] were detected at the beginning (*HvARF-like*) or end of the dark period (*HvOXS2*, *HvmTREF*) under W light in the normal light/dark cycle. Their delayed peaking compared to that of the circadian clock genes might be a sign of a regulatory interaction; however, this possible control does not exist in constant light as shown by continuous low expression levels of the redox-related transcription factors. Both the timing of oscillations and maximum level of their transcripts were modified by a low R/FR ratio or supplemental B light, which corroborates the importance of the spectral composition in the transcriptional control of the redox environment. The large induction of *HvARF* and *HvmTREF* expression by a low R/FR ratio, as observed for *HvTOC1*, may indicate their coordinated adjustment in light/dark cycles.

Concerning nitrate and sulfate assimilation, both processes must be synchronized with the photoperiod since photosynthesis ensures the energy and reductants for these pathways. The activation of the studied redox-responsive transcription factors during the night may contribute—through their involvement in the redox signaling—to the subsequent induction of the redox-dependent nitrate and sulfate reductions. In accordance with this hypothesis, the maximum transcript levels of *HvNR* and *HvAPSR*, encoding the key enzymes of the two pathways, were detected during dawn and in the first half of the light period, respectively (Figure 4a and Figure 5a). Correspondingly, the maximum activities of these two enzymes were detected later during the day, in the second half of the light period during their diurnal changes in maize [37]. While the circadian oscillations in nitrate and sulfate reduction in W light were characterized earlier [42,60], their regulation by the changes in the B and R/FR spectral components has not been studied before. The effect of R/FR light on *NR* mRNA and activity level was studied at a certain sampling point [61] but not in a time course during the whole day. The control of the daily oscillations in both *HvNR* and *HvAPSR* transcripts by supplemental FR and B light (especially in continuous illumination) was demonstrated in the present study since the timing and magnitude of the maximum expressions changed compared to W light.

Following nitrate reduction, nitrogen will be incorporated first into Gln, and the light-dependent synchronization of nitrate reduction and Gln synthesis was observed based on the maximum *HvNR*, *HvGLNb* and *HvGLNd* expressions during dawn. The activity of the encoded enzymes also increased simultaneously during the subsequent light period in barley [62]. The transcriptional coordination of these enzymes existed only in white light but disappeared under low R/FR ratio or additional B-light conditions, since the oscillations in the three transcripts were affected differently by the modification of the spectrum, especially in constant light. Interestingly, expression patterns of *HvGLNa* and *HvGLNe* were modified in a different way by the light spectrum compared to *HvGLNb* and *HvGLNd*, since only the peaks of the former ones were much greater in B light than in the white one. This difference can allow the specific adjustment of Gln formation under various environmental conditions.

Based on the diurnal changes in the level of transcripts related to sulfate reduction (*HvAPSR)* and GSH synthesis (*Hvγ-ECS)* in barley leaves and in the glutathione content in spruce needles [63], a clear diurnal cycling of Cys and GSH contents was expected in barley, but only their smaller oscillations were observed. This contradiction can be explained by the post-transcriptional control of Cys and GSH synthesis based on the regulation of the number of mRNAs (miRNA-associated degradation) and proteins, as well as enzyme activities. In addition, species-dependent differences in the diurnal oscillation of GSH levels can occur taking into account the comparison of the results obtained in spruce and barley. The changes in the size of the glutathione pool (GSH+GSSG) and the GSSG/GSH ratio resulted in a daily oscillation in the half-cell reduction potential with an amplitude of about 20 mV (Supplementary Figure S3), which can greatly influence the function of redox-sensitive proteins. As observed earlier in wheat [64], a moderate increase could be detected in the GSH content during the light period, and high expression of *HvAPSR* in the meantime could contribute to this change by allowing a higher rate of Cys formation during the sulfate reduction and providing sufficient Cys for GSH synthesis. The low R/FR ratio increased both the expression of *HvAPSR* and *Hvγ-ECS* and the amount of Cys and GSH, indicating the spectral control of these genes and metabolites. The effect of the light spectrum on GSH content was studied earlier in wheat, where supplemental B light also resulted in a higher level of GSH content compared to W light [65]. Similarly to GSH content, an increase in the expression of *HvGST* and *HvGR* was observed during the light period. Accordingly, the activities of the encoded enzymes peaked during the first part of the light period in maize [37]. These enzymes use GSH for the removal of toxic peroxides produced in greater amount during illumination of the plants [4,66]. The expression of these genes, together with the GSH concentration, was far higher in supplemental FR light then in W light, especially under constant illumination. The effect of the spectrum on GSTs was shown both at transcript level and on the enzymatic activity in various plant species (see for review [4]).

The expression of antioxidant-enzyme-coding genes generally increased (except for *HvCAT1*) during the dark period. Thus, the GSSG accumulating during the light period can be regenerated by ascorbate peroxidase in the dark. High transcript levels of *HvCAT2*, *HvCAT4* and *HvPRX* in cycling W light possibly cause a subsequent increase in the enzyme activity during the light period, as observed previously in wheat and *Arabidopsis* [30,64]. As described for *HvGST* and *HvGR*, maximum transcript levels of *HvAPX1*, *HvAPX4, HvCAT2*, *HvCAT4* and *HvPRX1* were also greater in low R/FR compared to W and supplemental B light during light/dark cycles, which further confirms the coordinated regulation of the various antioxidant genes by R and FR lights. The regulation of *GR*, *GST* and *APX* genes by R, FR and B lights was also observed in wheat [65]. In constant light however, the greatest transcript levels of *HvCAT4* and *HvPRX1* genes were observed in supplemental B light. Thus, the various spectral components have different effects on the levels and oscillations in the gene expression in cycling light than in constant light.

The results discussed above confirm the general regulatory effect of the circadian clock on the activity of genes related to different metabolic- and redox-responsive processes. Mutations in *CCA1*, *ELF3* and *LUX* affected the transcriptional regulation of ROS-responsive genes; therefore, circadian core genes may regulate phase-specific expression of these genes to allow the anticipation of oxidative stress according to a diurnal rhythm [30,67]. ROS functions also affect the transcriptional output of the clock, suggesting that the cellular redox state and circadian rhythms continuously interact and are affected by environmental factors [58,67,68]. A direct regulatory relationship between *HvCCA1* and *HvAPX4*, *HvGSH1*, *HvCAT1* and *HvCAT3* may be present based on the results in *Arabidopsis* in which CCA1-binding elements are present in the promoter of the four latter genes [30].

**Figure 7:**
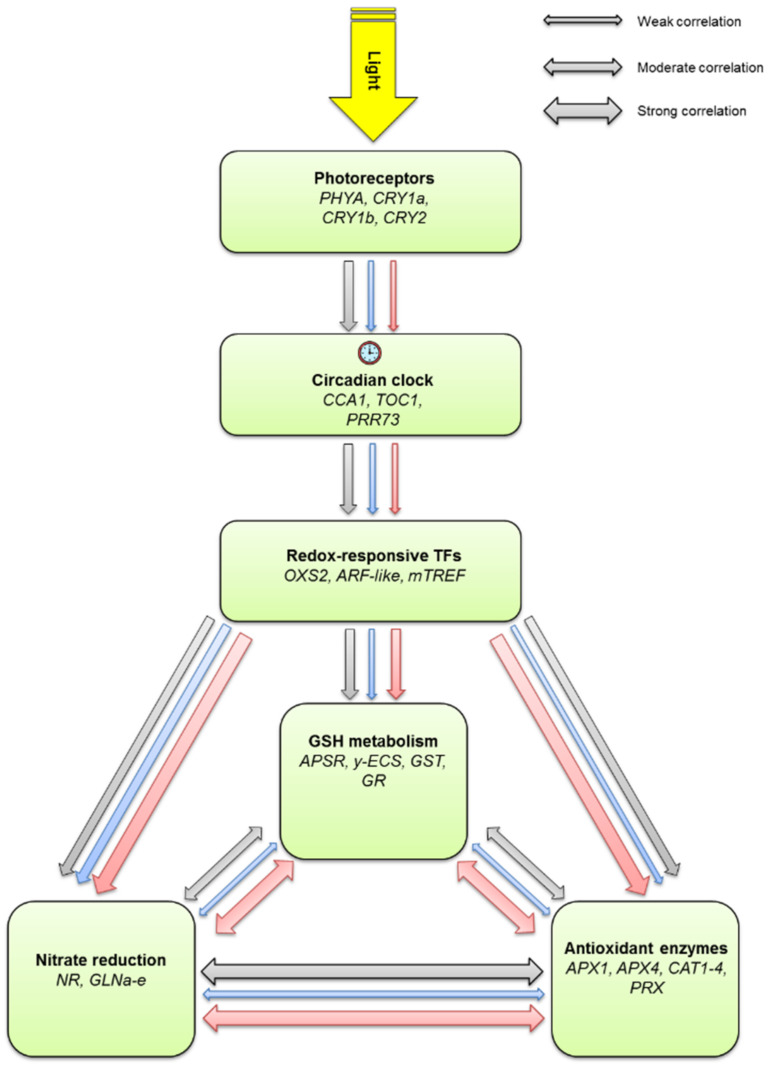
Putative spectral control of the diurnal changes in the transcription of the genes related to nitrate reduction, glutathione metabolism and antioxidant enzymes based on the correlation analysis. Green squares indicate the groups of evaluated genes related to different processes, one-way arrows show hypothetical regulatory effect, while duplex arrows indicate possible interactions between the different metabolic pathways. Three different arrow thicknesses indicate the level of correlation (weak, moderate and strong), spectral effect is represented by gray (normal white light), blue (supplemental blue light) and red (supplemental far-red light) colors. Correlation analysis was performed according to [69]. The correlation between different groups was considered “strong” if there was at least one pair of genes with “very high” (0.90–1.00) or “moderate” correlation, if there was at least one pair with “high” (0.70–0.90) correlation value and “weak” if correlation values were less than 0.7 (for details, see Supplementary Tables S1–S3).

A model for the spectrum-dependent control of the diurnal changes in the transcripts related to nitrate reduction, glutathione metabolism and antioxidant enzymes was created based on the correlations of gene expressions in normal W and supplemental B and FR light (Figure 7). In W light, there was a moderate positive correlation between the diurnal changes in most compared transcript levels except for the strong positive correlation between the genes associated with nitrate reduction and antioxidant enzymes. Interestingly, a similar weak correlation was found between the expression patterns of the neighboring components related to the proposed regulatory pathways from the photoreceptors and the redox-responsive transcription factors in B and FR light, indicating a balanced effect of the individual spectral components. However, in the downstream part of the network, the correlation was in general strong for FR light, weak for B light and moderate for W light, which indicates a major role of the FR spectral component in the transcriptional coordination of the diurnal rhythm of nitrate reduction, glutathione metabolism and antioxidant enzymes. Its ratio is high during dawn and dusk when the solar elevation angle is low [7] and the photosynthesis will be started and finished. Thus, the available energy and reducing power, as well the possible formation of ROS, greatly changes; therefore, the synchronized adjustment of the above listed biochemical processes is important.

## 4. Materials and Methods

### 4.1. Plant Material and Growth Conditions

The cold-tolerant, winter-habit barley (*Hordeum vulgare* spp. *vulgare*) variety, Nure, was used in this experiment in order to exclude the effect of the vegetative–generative transition on the studied parameters. This genotype needs vernalization (growth at low temperature) for the transition. After germination in Petri dishes between wet (distilled H_2_O) filter papers for 1 day at 25 °C and for 3 days at 4 °C, seedlings were planted into wooden boxes in a 2:1:1 (*v*/*v*/*v*) mixture of soil, sand and humus. The plantlets were grown in plant growth chambers (Conviron PGV36; Controlled Environments Ltd.; Winnipeg, MB, Canada) with 12 h photoperiods, at 20 °C/17 °C (day/night), with 70–75% RH, at 250 µmol m^−2^ s^−1^ light intensity under cool white fluorescent tubes (Sylvania 215 W F96 T12) for 7 days and subsequently at steady 22 °C for 5 days without changing the other cultivation conditions.

### 4.2. Light Treatment and Sampling

One-third of the 16-day-old plants started to receive supplemental far-red (FR) light treatment and one-third of them supplemental blue (B) light treatment in the developmental phase Z13 on the Zadoks scale when there were three fully developed leaves [70]. The remaining plants were further treated with fluorescent white light only and considered as controls. Plants were inside the same growth chamber with white separators between the treatments to prevent light contamination. Treated and control plants were subjected to the same environmental factors during the whole experiment except for the supplemental monochromatic light treatments. The sampling started after 7 days of light treatment. On the 1st and 2nd days, a 12 h photoperiod was applied, while on the 3rd and 4th days, the experiments were conducted under constant-light conditions. The supplemental FR (735 nm) and B (450 nm) lights were added to the fluorescent white light by 3 W high-power LED panels (Shenzhen Justar Electronic Technology Co., Ltd., Shenzhen, China) producing 500 μW/cm^2^ (about 22 µmol m^−2^ s^−1^). Thus, the 250 µmol m^−2^s^−1^ light intensity of the white fluorescent tubes was only increased by 9%, and it was not changed substantially. Based on [1], we used the lowest R/FR ratio, which provided a stable 0.5 R/FR ratio in our plant growth chamber. The light intensity was not changed during the experiment. Three independent experiments were carried out.

After one week of supplemental FR- and B-light treatments, leaf samples were collected every 4 h during the following 4 days. The plants reached the developmental stage Z14 (23 days old, four fully developed leaves, vegetative stage) when the sampling was started. The youngest fully developed leaves (the fourth ones from the stem bottom) were collected for biochemical analyses. The very first sample was collected immediately after the light switched on, i.e., at the beginning of the 8th day of the light treatment. At every sampling time, leaves were collected from the FR- or B-light-treated plants and the control plants as well; each sample consisted of a mixture of leaves from three–five independent plants, which were pooled and homogenized for RNA isolation and thiol analysis.

### 4.3. Gene Expression Studies

The total RNA was isolated by Direct-zolTM RNA MiniPrep kit (Zymo Research Corp., Irvine, CA, USA) from the leaves according to the manufacturer’s instructions, and then RNA quality was checked by a NanoDrop 2000 Spectrophotometer (Thermo Fisher Scientific Inc., Wilmington, MA, USA). Approx. 1 µg of total RNA was reverse-transcribed using the M-MLV Reverse Transcriptase and oligo(dT)15 primer (Promega Corporation, Madison, WI, USA) according to the manufacturer’s protocol. The gene expression levels were determined with the CFX96 TouchTM Real-Time PCR Detection System (Bio-Rad Hungary Ltd., Budapest, Hungary) using the KAPA SYBR^®^ FAST Master Mix (2×) Universal qPCR Kit (Kapa Biosystems, Inc., Wilmington, MA, USA) following the supplier’s instructions. The relative gene expressions were calculated using the ΔCt method [71] for which Ct values were normalized by the Ct values of *HvCyclophilin* since its transcript level was not affected by the treatments, and in our previous works it proved to be a reliable reference gene in barley [2,3].

The selection of the examined genes was carried out using a 3-way approach (Supplementary Figure S1). First of all, we collected redox/circadian-associated candidate genes from the corresponding literature [59,72,73]. Secondly, the *Hordeum vulgare* reference proteome was retrieved from Ensembl Plants FTP server (ftp.ensemblgenomes.org; release-41). The whole protein collection was subsequently scanned with the Hidden Markov Model (HMM)-based HMMER version 3.0 software package http://eddylab.org/software/hmmer/) [74] using redox- and circadian-related HMM profiles of the Pfam 32.0 database (ftp://ftp.ebi.ac.uk) [75]. Thirdly, we performed selection of genes responsive to far-red light with high expression changes (Galiba and Cattivelli, unpublished RNA-seq data). The primers designed for the selected sequences are listed in Supplementary Table S4. The correlation analysis between the transcription profile of the investigated genes was performed by the method of [69]. Graphical visualization of the expression studies of the investigated genes was performed with Origin 2018 software package (https://www.originlab.com/2018).

### 4.4. Determination of Thiol Content

The plant material was ground with liquid nitrogen in a mortar, after which 1 mL of 0.1 M HCl was added to the 200 mg plant sample. The determination of total and oxidized thiol content and the reduction in oxidized thiols were performed according to [76]. The numbers of reduced thiols were calculated as the difference between the numbers of total and oxidized thiols. The half-cell reduction potential of the thiol redox couples was calculated using the Nernst equation [77].

### 4.5. Statistical Analysis

The statistical evaluation of the data was carried out by analysis of variance and correlation using IBM-SPSS statistics software. The least significant difference (LSD) test was carried out to compare the mean values at 5% probability level.

## 5. Conclusions

Our results indicate the importance of the photoperiod and light spectrum in the control of the most studied redox-related transcripts and thiols since the changes in their levels exhibited a circadian or diurnal rhythm and were affected by both supplemental FR and B light. The various spectral components have special roles in the regulation of several of them as shown by the inducing effect of FR light and the inhibitory effect of B light in light/dark cycles and the activating influence of B light in constant illumination. The simultaneous or shifted oscillations in the expressions of the genes encoding enzymes of sulfate and nitrate reductions and maintenance of H_2_O_2_ levels may indicate their coordination by light spectrum, which is important in the adaptation to the altered environmental conditions during day/night and at high altitudes or latitudes. The correlation analysis of the diurnal changes in the investigated transcript levels indicated a major role of far-red light in this coordination. The different regulation of certain studied transcripts in constant light compared to light/dark cycles indicated the lack of the circadian, light-dependent entrainment of the oscillations in their level, and it may be also based on the different availability of the reducing power and energy under the two types of illuminations. On the other hand, the circadian control of other genes, masked by the possibility of the direct light induction in cycling light, was confirmed in constant light. An interesting future research direction could be the study of the possible redox- and light-associated diurnal/circadian control of the amount/activity of proteins encoded by the investigated genes.

## Figures and Tables

**Table 1:** Type of oscillations in transcript and thiol levels and half-cell reduction potentials under various spectral conditions.

	Parameter	White	Far-Red	Blue
**Photoreceptors**	*HvPHYA*	irregular	* <circadian	<diurnal
	*HvCRY1a*	diurnal	<diurnal	=diurnal
	*HvCRY1b*	diurnal	<diurnal	=diurnal
	*HvCRY2*	irregular	<diurnal	=diurnal
**Circadian clock**	*HvCCA1*	circadian	<diurnal	<circadian
	*HvTOC1*	circadian	<diurnal	<circadian
	*HvPRR73*	circadian	>diurnal	>circadian
**Redox-responsive TFs**	*HvOXS2*	diurnal	=diurnal	<irregular
	*HvARF-like*	circadian	<circadian	<circadian
	*HvmTREF*	diurnal	<diurnal	=diurnal
**Nitrate reduction**	*HvNR*	circadian	<diurnal	<circadian
	*HvGLNa*	irregular	<irregular	<irregular
	*HvGLNb*	diurnal	=diurnal	<irregular
	*HvGLNd*	diurnal	<diurnal	=no
	*HvGLNe*	no	=no	<irregular
**Glutathione metabolism**	*HvAPSR*	diurnal	<irregular	<circadian
	*Hvγ-ECS*	diurnal	<irregular	>irregular
	*HvGST*	circadian	>circadian	=circadian
	*HvGR*	circadian	>diurnal	<irregular
**Antioxidant enzymes**	*HvAPX1*	diurnal	>diurnal	<diurnal
	*HvAPX4*	irregulal	>irregular	<no
	*HvCAT1*	circadian	=circadian	=circadian
	*HvCAT2*	diurnal	>irregular	>irregular
	*HvCAT4*	diurnal	>irregular	>diurnal
	*HvPRX*	irregular	>irregular	>irregular
**Thiols**	Cys	irregular	<irregular	>irregular
	CySS	irregular	> irregular	<irregular
	E_CySS/Cys_	no	>no	<no
	GSH	irregular	<irregular	>irregular
	GSSG	irregular	=irregular	<irregular
	E_GSSG/GSH_	no	>no	<no

* The signs <, > and = indicate whether the investigated parameters were greater, smaller or equal in far-red or blue light compared to white light, respectively. Gray background shows that the levels increased during the dark phase and peaked at the latest phase during dawn. TF: transcription factor, E: half-cell reduction potential.

## Data Availability

Not applicable.

## Supplementary Materials

The following supporting information can be downloaded at: https://www.mdpi.com/article/10.3390/ijms23137479/s1. References [69,74,75,78] are cited in the Supplementary Materials.

## References

[1] Franklin K.A., Whitelam G.C. (2007). Light-Quality Regulation of Freezing Tolerance in Arabidopsis Thaliana. Nat. Genet..

[2] Novák A., Boldizsár Á., Ádám É., Kozma-Bognár L., Majláth I., Båga M., Tóth B., Chibbar R., Galiba G. (2016). Light-Quality and Temperature-Dependent *CBF14* Gene Expression Modulates Freezing Tolerance in Cereals. EXBOTJ.

[3] Gierczik K., Novák A., Ahres M., Székely A., Soltész A., Boldizsár Á., Gulyás Z., Kalapos B., Monostori I., Kozma-Bognár L. (2017). Circadian and Light Regulated Expression of CBFs and Their Upstream Signalling Genes in Barley. IJMS.

[4] Gallé Á., Czékus Z., Bela K., Horváth E., Ördög A., Csiszár J., Poór P. (2019). Plant Glutathione Transferases and Light. Front. Plant Sci..

[5] Somers D.E., Devlin P.F., Kay S.A. (1998). Phytochromes and Cryptochromes in the Entrainment of the *Arabidopsis* Circadian Clock. Science.

[6] Cashmore A.R., Jarillo J.A., Wu Y.-J., Liu D. (1999). Cryptochromes: Blue Light Receptors for Plants and Animals. Science.

[7] Chiang C., Olsen J.E., Basler D., Bånkestad D., Hoch G. (2019). Latitude and Weather Influences on Sun Light Quality and the Relationship to Tree Growth. Forests.

[8] Hotta C.T., Gardner M.J., Hubbard K.E., Baek S.J., Dalchau N., Suhita D., Dodd A.N., Webb A.A.R. (2007). Modulation of Environmental Responses of Plants by Circadian Clocks: Circadian Modulation of Plant Environmental Responses. Plant Cell Environ..

[9] Covington M.F., Maloof J.N., Straume M., Kay S.A., Harmer S.L. (2008). Global Transcriptome Analysis Reveals Circadian Regulation of Key Pathways in Plant Growth and Development. Genome Biol..

[10] Campoli C., Shtaya M., Davis S.J., Von Korff M. (2012). Expression Conservation within the Circadian Clock of a Monocot: Natural Variation at Barley Ppd-H1 Affects Circadian Expression of Flowering Time Genes, but Not Clock Orthologs. BMC Plant Biol..

[11] Franklin K.A., Toledo-Ortiz G., Pyott D.E., Halliday K.J. (2014). Interaction of Light and Temperature Signalling. J. Exp. Bot..

[12] Grundy J., Stoker C., Carré I.A. (2015). Circadian Regulation of Abiotic Stress Tolerance in Plants. Front. Plant Sci..

[13] Wang Z.-Y., Tobin E.M. (1998). Constitutive Expression of the CIRCADIAN CLOCK ASSOCIATED 1 (CCA1) Gene Disrupts Circadian Rhythms and Suppresses Its Own Expression. Cell.

[14] Alabadi D. (2001). Reciprocal Regulation Between TOC1 and LHY/CCA1 Within the Arabidopsis Circadian Clock. Science.

[15] Nohales M.A., Kay S.A. (2016). Molecular Mechanisms at the Core of the Plant Circadian Oscillator. Nat. Struct. Mol. Biol..

[16] Murakami M., Tago Y., Yamashino T., Mizuno T. (2007). Comparative Overviews of Clock-Associated Genes of Arabidopsis Thaliana and Oryza Sativa. Plant Cell Physiol..

[17] Murakami M., Ashikari M., Miura K., Yamashino T., Mizuno T. (2003). The Evolutionarily Conserved OsPRR Quintet: Rice Pseudo-Response Regulators Implicated in Circadian Rhythm. Plant Cell Physiol..

[18] Decousset L., Griffiths S., Dunford R.P., Pratchett N., Laurie D.A. (2000). Development of STS Markers Closely Linked to the Ppd-H1 Photoperiod Response Gene of Barley (*Hordeum vulgare* L.). Theor. Appl. Genet..

[19] Turner A. (2005). The Pseudo-Response Regulator Ppd-H1 Provides Adaptation to Photoperiod in Barley. Science.

[20] Miwa K., Serikawa M., Suzuki S., Kondo T., Oyama T. (2006). Conserved Expression Profiles of Circadian Clock-Related Genes in Two Lemna Species Showing Long-Day and Short-Day Photoperiodic Flowering Responses. Plant Cell Physiol..

[21] Serikawa M., Miwa K., Kondo T., Oyama T. (2008). Functional Conservation of Clock-Related Genes in Flowering Plants: Overexpression and RNA Interference Analyses of the Circadian Rhythm in the Monocotyledon Lemna Gibba. Plant Physiol..

[22] Khan S., Rowe S.C., Harmon F.G. (2010). Coordination of the Maize Transcriptome by a Conserved Circadian Clock. BMC Plant Biol..

[23] Filichkin S.A., Breton G., Priest H.D., Dharmawardhana P., Jaiswal P., Fox S.E., Michael T.P., Chory J., Kay S.A., Mockler T.C. (2011). Global Profiling of Rice and Poplar Transcriptomes Highlights Key Conserved Circadian-Controlled Pathways and Cis-Regulatory Modules. PLoS ONE.

[24] Foyer C.H., Noctor G. (2005). Oxidant and Antioxidant Signalling in Plants—Reevaluation of the Concept. Plant Cell Environ..

[25] Kocsy G., Tari I., Vanková R., Zechmann B., Gulyás Z., Poór P., Galiba G. (2013). Redox Control of Plant Growth and Development. Plant Sci..

[26] Apel K., Hirt H. (2004). REACTIVE OXYGEN SPECIES: Metabolism, Oxidative Stress, and Signal Transduction. Annu. Rev. Plant Biol..

[27] Boldt R., Scandalios J.G. (1997). Influence of UV-Light on the Expression of the Cat2 and Cat3 Catalase Genes in Maize. Free Radic. Biol. Med..

[28] Zhong H.H., McClung C.R. (1996). The Circadian Clock Gates Expression of Two Arabidopsis Catalase Genes to Distinct and Opposite Circadian Phases. Mol. Gen. Genet..

[29] Horling F., Lamkemeyer P., König J., Finkemeier I., Kandlbinder A., Baier M., Dietz K.-J. (2003). Divergent Light-, Ascorbate-, and Oxidative Stress-Dependent Regulation of Expression of the Peroxiredoxin Gene Family in Arabidopsis. Plant Physiol..

[30] Lai A.G., Doherty C.J., Mueller-Roeber B., Kay S.A., Schippers J.H.M., Dijkwel P.P. (2012). CIRCADIAN CLOCK-ASSOCIATED 1 Regulates ROS Homeostasis and Oxidative Stress Responses. Proc. Natl. Acad. Sci. USA.

[31] Edgar R.S., Green E.W., Zhao Y., Van Ooijen G., Olmedo M., Qin X., Xu Y., Pan M., Valekunja U.K., Feeney K.A. (2012). Peroxiredoxins Are Conserved Markers of Circadian Rhythms. Nature.

[32] Kubo A., Saji H., Tanaka K., Kondo N. (1995). Expression of Arabidopsis Cytosolic Ascorbate Peroxidase Gene in Response to Ozone or Sulfur Dioxide. Plant Mol. Biol..

[33] Agrawal G.K., Jwa N.-S., Iwahashi H., Rakwal R. (2003). Importance of Ascorbate Peroxidases OsAPX1 and OsAPX2 in the Rice Pathogen Response Pathways and Growth and Reproduction Revealed by Their Transcriptional Profiling. Gene.

[34] Meyer A.J. (2008). The Integration of Glutathione Homeostasis and Redox Signaling. J. Plant Physiol..

[35] Anjum N.A., Umar S., Chan M.T. (2010). Ascorbate-Glutathione Pathway and Stress Tolerance in Plants.

[36] Caverzan A., Passaia G., Rosa S.B., Ribeiro C.W., Lazzarotto F., Margis-Pinheiro M. (2012). Plant Responses to Stresses: Role of Ascorbate Peroxidase in the Antioxidant Protection. Genet. Mol. Biol..

[37] Kocsy G., Owttrim G., Brander K., Brunold C. (1997). Effect of Chilling on the Diurnal Rhythm of Enzymes Involved in Protection against Oxidative Stress in a Chilling-Tolerant and a Chilling-Sensitive Maize Genotype. Physiol. Plant..

[38] Kopriva S., Muheim R., Koprivova A., Trachsel N., Catalano C., Suter M., Brunold C. (1999). Light Regulation of Assimilatory Sulphate Reduction in Arabidopsis Thaliana. Plant J..

[39] Kopriva S., Rennenberg H. (2004). Control of Sulphate Assimilation and Glutathione Synthesis: Interaction with N and C Metabolism. J. Exp. Bot..

[40] Hesse H., Nikiforova V., Gakiere B., Hoefgen R. (2004). Molecular Analysis and Control of Cysteine Biosynthesis: Integration of Nitrogen and Sulphur Metabolism. J. Exp. Bot..

[41] Lillo C. (1994). Light Regulation of Nitrate Reductase in Green Leaves of Higher Plants. Physiol. Plant..

[42] Lillo C., Meyer C., Ruoff P. (2001). The Nitrate Reductase Circadian System. The Central Clock Dogma Contra Multiple Oscillatory Feedback Loops: Figure 1. Plant Physiol..

[43] Carvalho R.F., Campos M.L., Azevedo R.A., Ahmad P., Azooz M.M., Prasad M.N.V. (2013). The role of phytochromes in stress tolerance. Salt Stress in Plants: Signalling, Omics and Adaptations.

[44] Consentino L., Lambert S., Martino C., Jourdan N., Bouchet P.-E., Witczak J., Castello P., El-Esawi M., Corbineau F., D’Harlingue A. (2015). Blue-Light Dependent Reactive Oxygen Species Formation by Arabidopsis Cryptochrome May Define a Novel Evolutionarily Conserved Signaling Mechanism. New Phytol..

[45] Jourdan N., Martino C.F., El-Esawi M., Witczak J., Bouchet P.-E., D’Harlingue A., Ahmad M. (2015). Blue-Light Dependent ROS Formation by Arabidopsis Cryptochrome-2 May Contribute toward Its Signaling Role. Plant Signal. Behav..

[46] Gavassi M.A., Monteiro C.C., Campos M.L., Melo H.C., Carvalho R.F. (2017). Phytochromes Are Key Regulators of Abiotic Stress Responses in Tomato. Sci. Hortic..

[47] El-Esawi M., Arthaut L.-D., Jourdan N., D’Harlingue A., Link J., Martino C.F., Ahmad M. (2017). Blue-Light Induced Biosynthesis of ROS Contributes to the Signaling Mechanism of Arabidopsis Cryptochrome. Sci. Rep..

[48] D’Amico-Damião V., Carvalho R.F. (2018). Cryptochrome-Related Abiotic Stress Responses in Plants. Front. Plant Sci..

[49] Dutta Gupta S., Agarwal A., Dutta Gupta S. (2017). Influence of LED Lighting on In Vitro Plant Regeneration and Associated Cellular Redox Balance. Light Emitting Diodes for Agriculture.

[50] Shohael A.M., Ali M.B., Yu K.W., Hahn E.J., Islam R., Paek K.Y. (2006). Effect of Light on Oxidative Stress, Secondary Metabolites and Induction of Antioxidant Enzymes in Eleutherococcus Senticosus Somatic Embryos in Bioreactor. Process Biochem..

[51] Baque M.A., Hahn E.-J., Paek K.-Y. (2010). Induction Mechanism of Adventitious Root from Leaf Explants of Morinda Citrifolia as Affected by Auxin and Light Quality. In Vitro Cell. Dev. Biol. Plant.

[52] Bartoli C.G., Tambussi E.A., Diego F., Foyer C.H. (2009). Control of Ascorbic Acid Synthesis and Accumulation and Glutathione by the Incident Light Red/Far Red Ratio in *Phaseolus vulgaris* Leaves. FEBS Lett..

[53] Cameron J.C., Pakrasi H.B. (2010). Essential Role of Glutathione in Acclimation to Environmental and Redox Perturbations in the Cyanobacterium *Synechocystis* sp. PCC 6803. Plant Physiol..

[54] Devlin P.F., Kay S.A. (2000). Cryptochromes Are Required for Phytochrome Signaling to the Circadian Clock but Not for Rhythmicity. Plant Cell.

[55] Yanovsky M.J., Kay S.A. (2003). Living by the Calendar: How Plants Know When to Flower. Nat. Rev. Mol. Cell Biol..

[56] Salomé P.A., Mcclung C.R. (2005). What Makes the Arabidopsis Clock Tick on Time? A Review on Entrainment. Plant Cell Environ..

[57] Deng W., Clausen J., Boden S., Oliver S.N., Casao M.C., Ford B., Anderssen R.S., Trevaskis B. (2015). Dawn and Dusk Set States of the Circadian Oscillator in Sprouting Barley (*Hordeum vulgare*) Seedlings. PLoS ONE.

[58] Yakir E., Hilman D., Harir Y., Green R.M. (2007). Regulation of Output from the Plant Circadian Clock. FEBS J..

[59] Matsuo M., Johnson J.M., Hieno A., Tokizawa M., Nomoto M., Tada Y., Godfrey R., Obokata J., Sherameti I., Yamamoto Y.Y. (2015). High REDOX RESPONSIVE TRANSCRIPTION FACTOR1 Levels Result in Accumulation of Reactive Oxygen Species in Arabidopsis Thaliana Shoots and Roots. Mol. Plant.

[60] Leustek T. (2002). Sulfate Metabolism. Arab. Book.

[61] Lillo C., Appenroth K.-J. (2001). Light Regulation of Nitrate Reductase in Higher Plants: Which Photoreceptors Are Involved?. Plant Biol..

[62] Lillo C. (1984). Diurnal Variations of Nitrite Reductase, Glutamine Synthetase, Glutamate Synthase, Alanine Aminotransferase and Aspartate Aminotransferase in Barley Leaves. Physiol. Plant.

[63] Schupp R., Rennenberg H. (1988). Diurnal Changes in the Glutathione Content of Spruce Needles (*Picea abies* L.). Plant Sci..

[64] Cao J., Gulyás Z., Kalapos B., Boldizsár Á., Liu X., Pál M., Yao Y., Galiba G., Kocsy G. (2019). Identification of a Redox-Dependent Regulatory Network of MiRNAs and Their Targets in Wheat. J. Exp. Bot..

[65] Toldi D., Gyugos M., Darkó É., Szalai G., Gulyás Z., Gierczik K., Székely A., Boldizsár Á., Galiba G., Müller M. (2019). Light Intensity and Spectrum Affect Metabolism of Glutathione and Amino Acids at Transcriptional Level. PLoS ONE.

[66] Jiang H.-W., Liu M.-J., Chen I.-C., Huang C.-H., Chao L.-Y., Hsieh H.-L. (2010). A Glutathione S-Transferase Regulated by Light and Hormones Participates in the Modulation of Arabidopsis Seedling Development. Plant Physiol..

[67] Zhou M., Wang W., Karapetyan S., Mwimba M., Marqués J., Buchler N.E., Dong X. (2015). Redox Rhythm Reinforces the Circadian Clock to Gate Immune Response. Nature.

[68] De Montaigu A., Tóth R., Coupland G. (2010). Plant Development Goes like Clockwork. Trends Genet..

[69] Guilford J.P. (1950). Creativity. Am. Psychol..

[70] Zadoks J.C., Chang T.T., Konzak C.F. (1974). A Decimal Code for the Growth Stages of Cereals. Weed Res..

[71] Livak K.J., Schmittgen T.D. (2001). Analysis of Relative Gene Expression Data Using Real-Time Quantitative PCR and the 2−ΔΔCT Method. Methods.

[72] He H., Van Breusegem F., Mhamdi A. (2018). Redox-Dependent Control of Nuclear Transcription in Plants. J. Exp. Bot..

[73] De Carvalho Oliveira R.A., De Andrade A.S., Imparato D.O., De Lima J.G.S., De Almeida R.V.M., Lima J.P.M.S., Pasquali M.A., De Bittencourt Pasquali M.A., Dalmolin R.J.S. (2019). Analysis of Arabidopsis Thaliana Redox Gene Network Indicates Evolutionary Expansion of Class III Peroxidase in Plants. Sci. Rep..

[74] Eddy S.R. (2009). A New Generation of Homology Search Tools Based on Probabilistic Inference. Genome Informatics 2009, Proceedings of the 20th International Conference, Pacifico Yokohama, Japan, 14–16 December 2009.

[75] El-Gebali S., Mistry J., Bateman A., Eddy S.R., Luciani A., Potter S.C., Qureshi M., Richardson L.J., Salazar G.A., Smart A. (2019). The Pfam Protein Families Database in 2019. Nucleic Acids Res..

[76] Gulyás Z., Boldizsár Á., Novák A., Szalai G., Pál M., Galiba G., Kocsy G. (2014). Central Role of the Flowering Repressor ZCCT2 in the Redox Control of Freezing Tolerance and the Initial Development of Flower Primordia in Wheat. BMC Plant Biol..

[77] Schafer F.Q., Buettner G.R. (2001). Redox Environment of the Cell as Viewed through the Redox State of the Glutathione Disulfide/Glutathione Couple. Free Radic. Biol. Med..

[78] Morran S., Eini O., Pyvovarenko T., Parent B., Singh R., Ismagul A., Eliby S., Shirley N., Langridge P., Lopato S. (2011). Improvement of stress tolerance of wheat and barley by modulation of expression of DREB/CBF factors. Plant Biotechnol. J..

