# Supplementary material for: Far-Red Light Coordinates the Diurnal Changes in the Transcripts Related to Nitrate Reduction, Glutathione Metabolism and Antioxidant Enzymes in Barley
Source: Int J Mol Sci. 2022 Jul 5;23(13):7479. doi: 10.3390/ijms23137479 (PMC9267158; doi:10.3390/ijms23137479)
Supplement: Supplementary file 1 [file ijms-23-07479-s001.zip › ijms-1776321-supplementary.pdf]

International Journal of *Molecular Sciences*

MDPI

Supplementary materials

Table S1. Correlation values according to Guilford [69] between the transcription level of the evaluate genes related to different processes under normal white light.

	**Table S1**	**Photoreceptors**				**Circadian clock**			**Redox-responsive TFs**			**Nitrate reduction**					**Glutathione metabolism**				**Antioxidant enzymes**						**Thiols**					
		**PhyA**	**Cry1a**	**Cry1b**	**Cry2**	**CCA1**	**TOC1**	**PRR73**	**OXS2**	**ARF-like**	**mTREF**	**NR**	**GLNa**	**GLNb**	**GLNd**	**GLNe**	**APSR**	**γ-ECS**	**GST**	**GR**	**APX1**	**APX4**	**CAT1**	**CAT2**	**CAT4**	**PRX**	**CyS**	**CySS**	**ECyS/CySS**	**GSH**	**GSSG**	**EGSH/GSSG**
**Photoreceptors**	**PhyA**		0.20	0.08	0.36	-0.22	0.70	-0.06	0.16	0.41	-0.12	-0.06	-0.11	0.17	0.14	0.39	0.09	0.37	0.22	0.60	0.11	-0.02	0.51	0.36	-0.17	0.04	-0.18	0.19	0.14	-0.10	0.23	0.11
	**Cry1a**			0.95	-0.06	0.54	0.06	-0.05	0.47	0.16	0.84	0.74	0.14	0.61	0.71	0.02	0.06	0.33	0.24	0.16	0.67	0.50	-0.01	0.31	0.62	0.16	0.06	-0.25	-0.18	-0.20	-0.27	0.07
	**Cry1b**				-0.	0.68	-0.04	-0.01	0.50	0.15	0.89	0.79	0.13	0.64	0.71	-0.01	0.06	0.30	0.10	0.12	0.67	0.51	-0.07	0.30	0.67	0.08	0.21	-0.38	-0.29	-0.	-0.28	-0.02

					04																									11		
Circadian clock	**Cry2**					-0.28	0.45	0.52	-0.05	0.45	0.11	-0.30	0.05	-0.06	-0.02	0.41	0.18	0.23	0.35	0.58	-0.12	-0.05	0.67	0.18	-0.30	0.13	-0.10	0.21	0.11	-0.12	-0.07	0.11
	**CCA1**						-0.50	-0.19	0.23	-0.36	0.51	0.86	0.07	0.37	0.35	-0.21	-0.01	-0.08	-0.17	-0.30	0.37	0.26	-0.35	-0.04	0.66	-0.22	0.29	-0.53	-0.38	-0.08	-0.03	0.03
	**TOC1**							0.12	0.26	0.73	0.05	-0.29	0.06	0.27	0.36	0.54	0.16	0.68	0.20	0.83	0.26	0.28	0.67	0.55	-0.19	0.30	-0.25	0.21	0.32	-0.16	0.06	0.17
	**PRR73**								-0.08	0.41	0.10	-0.21	0.25	-0.25	-0.08	0.48	0.45	0.05	0.53	0.39	-0.17	-0.01	0.51	-0.11	-0.06	0.03	-0.02	0.28	0.18	0.06	-0.14	-0.09
Redox-respon-	**OXS2**									0.33	0.53	0.53	0.15	0.80	0.68	0.18	0.12	0.60	-0.31	0.46	0.75	0.42	0.02	0.70	0.53	0.41	0.10	-0.04	-0.09	-0.01	-0.12	-0.07

	**ARF-like**										0.26	-0.28	0.19	0.28	0.35	0.49	0.17	0.62	0.09	0.79	0.28	0.33	0.67	0.57	-0.14	0.15	0.20	0.08	-0.08	0.30	-0.27	-0.33
	**mTREF**											0.63	0.23	0.63	0.73	0.07	0.10	0.39	0.10	0.19	0.65	0.60	0.00	0.35	0.58	0.18	0.13	-0.30	-0.17	-0.18	-0.34	0.07
**Nitrate reduction**	**NR**												0.11	0.63	0.60	-0.12	0.10	0.16	-0.05	-0.11	0.64	0.41	-0.34	0.14	0.80	0.08	0.10	-0.29	-0.22	-0.23	0.02	0.16
	**GLNa**													0.27	0.35	0.65	0.25	0.44	0.17	0.37	0.36	0.62	0.30	0.29	0.48	0.44	-0.07	-0.02	-0.04	-0.15	-0.10	0.11
	**GLNb**														0.91	0.18	0.00	0.79	-0.26	0.39	0.93	0.74	-0.02	0.77	0.69	0.51	0.20	-0.34	-0.28	-0.12	-0.17	0.00
	**GLNd**															0.24	0.09	0.81	-0.	0.	0.95	0.86	0.07	0.70	0.72	0.	0.	-0.	-0.16	-0.	-0.	0.11

																		01	45							57	08	40		25	28	
	**GLNe**																0.37	0.57	0.30	0.71	0.15	0.40	0.75	0.39	0.12	0.25	-0.28	0.23	0.29	-0.26	0.07	0.20
**Glutathione metabolism**	**APSR**																	0.08	0.39	0.39	0.06	0.03	0.30	-0.06	0.19	0.04	-0.27	0.41	0.37	-0.17	0.41	0.25
	**γ-ECS**																		0.00	0.75	0.74	0.76	0.42	0.84	0.38	0.61	0.01	-0.15	-0.03	-0.17	-0.13	0.08
	**GST**																			0.23	-0.16	0.03	0.43	-0.17	-0.02	0.08	-0.44	0.31	0.39	-0.40	0.18	0.40
	**GR**																				0.39	0.36	0.79	0.67	0.07	0.43	-0.10	0.23	0.17	-0.07	0.02	0.06

Antioxidant enzymes	**APX1**																				0.82	-0.05	0.68	0.77	0.61	0.19	-0.35	-0.28	-0.10	-0.30	-0.03
	**APX4**																					0.07	0.56	0.63	0.53	0.07	-0.43	-0.18	-0.20	-0.32	0.07
	**CAT1**																						0.41	-0.17	0.09	-0.09	0.29	0.16	-0.03	-0.01	0.02
	**CAT2**																							0.34	0.50	0.20	-0.29	-0.25	0.03	-0.25	-0.14
	**CAT4**																								0.43	0.20	-0.33	-0.31	-0.14	-0.09	0.06
	**PRX**																									-0.[illegible]	0.06	0.03	-0.[illegible]	-0.14	0.15

																										15			23		
Thiols	CyS																										-0.48	-0.93	0.85	-0.46	-0.86
	CySS																											0.58	-0.12	0.47	0.22
	$E_{CyS/CySS}$																												-0.72	0.49	0.77
	GSH																													-0.33	-0.96
	GSSG																														0.53
	$E_{GSH/GSSG}$																														

* If correlation value is: < 0.20 - slight; almost negligible relationship; 0.20 - 0.40 - low correlation; definite but small relationship; 0.40 - 0.70 - moderate correlation; substantial relationship (green cells); 0.70 - 0.90 - high correlation; marked relationship (brown cells); 0.90 - 1.00 - very high correlation; very dependable relationship (black cells).

Table S2. Correlation values according to Guilford [69] between the transcription level of the evaluate genes related to different processes under additional blue light.

	Table S2	Photoreceptors				Circadian clock			Redox-responsive TFs			Nitrate reduction					Glutathione metabolism				Antioxidant enzymes						Thiols					
		PhyA	Cry1a	Cry1b	Cry2	CCA1	TOC1	PRR73	OXS2	ARF-like	mTREF	NR	GLNa	GLNb	GLNd	GLNe	APSR	γ-ECS	GST	GR	APX1	APX4	CAT1	CAT2	CAT4	PRX	CyS	CySS	$E_{CyS/CySS}$	GSH	GSSG	$E_{GSH/GSSG}$
Photoreceptors	PhyA		0.81	0.80	0.14	0.17	-0.10	-0.12	0.09	0.64	0.63	0.14	-0.55	0.34	0.36	0.15	-0.45	0.30	0.31	-0.28	0.23	-0.32	0.00	0.14	-0.40	-0.32	0.06	-0.30	-0.18	-0.33	-0.36	0.00
	Cry1a			0.98	0.01	0.54	-0.29	-0.09	0.04	0.31	0.75	0.26	-0.53	0.55	0.57	0.15	-0.47	0.24	0.35	-0.44	0.42	0.05	-0.21	0.02	-0.11	-0.34	-0.10	-0.29	0.02	-0.40	-0.31	0.12
	Cry1b				0.01	0.62	-0.34	-0.10	0.08	0.34	0.75	0.31	-0.49	0.57	0.57	0.16	-0.51	0.24	0.27	-0.38	0.47	0.11	-0.21	0.11	-0.10	-0.32	-0.13	-0.30	0.03	-0.42	-0.34	0.13
	Cry2					-0.32	0.33	0.65	-0.26	0.28	-0.12	-0.31	0.01	-0.29	-0.31	-0.20	-0.25	0.41	0.28	0.42	-0.41	-0.05	0.51	0.24	-0.66	-0.09	0.01	-0.13	-0.10	0.03	-0.07	-0.05
Circadian	CCA1						-0.58	-0.20	0.10	-0.35	0.33	0.49	-0.03	0.40	0.42	0.20	-0.27	-0.11	-0.08	-0.42	0.45	0.49	-0.45	-0.13	0.43	0.01	-0.17	-0.25	0.12	-0.45	-0.26	0.20

	**TOC1**							-0.06	0.24	0.16	-0.25	-0.03	-0.16	-0.04	-0.05	0.10	0.36	0.40	0.15	0.65	-0.28	-0.19	0.43	0.25	-0.47	-0.08	-0.07	0.32	0.19	0.28	0.37	-0.03
	**PRR73**								-0.41	0.08	-0.02	-0.30	0.36	-0.33	-0.33	-0.19	-0.12	0.05	0.36	0.10	-0.32	0.10	0.33	-0.07	-0.23	-0.20	0.05	0.18	-0.01	0.37	0.18	0.00
**Redox-responsive TFs**	**OXS2**									-0.06	-0.08	0.77	-0.09	0.57	0.44	0.80	0.25	0.48	-0.22	0.14	0.11	-0.06	-0.07	0.30	0.27	-0.05	-0.42	0.09	0.35	-0.22	0.14	0.31
	**ARF-like**										0.51	-0.19	-0.25	0.14	0.08	-0.14	-0.40	0.22	0.09	0.09	0.18	-0.25	0.38	0.34	-0.55	-0.26	0.08	-0.06	-0.14	0.10	-0.17	-0.16
	**mTREF**											0.03	-0.23	0.45	0.40	0.00	-0.48	0.04	0.06	-0.47	0.43	-0.07	0.09	-0.08	-0.11	-0.49	-0.06	-0.12	0.05	-0.22	-0.22	0.09
**Nitrate reduction**	**NR**												-0.11	0.71	0.70	0.92	0.21	0.24	-0.04	-0.09	0.15	0.21	-0.31	0.08	0.41	-0.06	-0.34	0.08	0.37	-0.26	0.05	0.23
	**GLNa**													-0.27	-0.25	-0.03	-0.04	-0.08	-0.32	0.12	-0.04	0.33	0.37	-0.13	0.34	0.07	-0.08	0.24	0.15	0.08	0.13	0.10
	**GLNb**														0.87	0.61	-0.26	0.48	-0.14	-0.26	0.60	0.21	-0.16	0.12	0.20	-0.29	-0.41	0.00	0.39	-0.38	-0.02	0.35
	**GLNd**															0.62	-0.24	0.35	0.11	-0.25	0.61	0.25	-0.28	0.00	0.28	-0.18	-0.28	0.10	0.35	-0.28	-0.02	0.19

	**GLNe**																0.26	0.36	-0.04	-0.09	0.02	-0.04	-0.10	0.01	0.34	-0.16	-0.42	0.22	0.44	-0.24	0.18	0.35
Glutathione metabolism	**APSR**																	-0.32	0.16	0.17	-0.53	-0.18	-0.01	-0.15	0.21	0.09	-0.10	0.36	0.19	0.29	0.37	-0.02
	**γ-ECS**																		0.01	0.29	0.10	-0.08	0.35	0.43	-0.31	-0.22	-0.27	-0.02	0.18	-0.15	0.06	0.24
	**GST**																			0.03	-0.25	-0.08	-0.05	-0.08	-0.17	-0.04	0.33	-0.03	-0.23	0.29	-0.05	-0.40
	**GR**																				-0.22	0.08	0.30	0.66	-0.29	0.22	-0.10	0.30	0.16	0.32	0.35	-0.03
Antioxidant enzymes	**APX1**																					0.47	-0.30	0.08	0.25	-0.12	-0.24	0.05	0.26	-0.24	-0.02	0.25
	**APX4**																						-0.24	0.04	0.38	0.02	-0.32	0.08	0.38	-0.06	0.07	0.17
	**CAT1**																							0.14	-0.48	-0.19	-0.18	0.15	0.12	-0.03	0.07	0.14
	**CAT2**																								-0.29	-0.14	-0.21	-0.02	0.04	-0.06	0.00	0.08

	**CAT4**																								0.18	-0.21	0.11	0.29	-0.04	0.11	0.14
	**PRX**																									0.28	-0.08	-0.20	0.10	-0.02	-0.23
Thiols	**CyS**																										-0.46	-0.90	0.30	-0.48	-0.88
	**CySS**																											0.73	0.52	0.92	0.48
	**$E_{CyS/CySS}$**																												-0.02	0.73	0.81
	**GSH**																													0.54	-0.35
	**GSSG**																														0.53
	**$E_{GSH/GSSG}$**																														

* If correlation value is: < 0.20 - slight; almost negligible relationship; 0.20 - 0.40 - low correlation; definite but small relationship; 0.40 - 0.70 - moderate correlation; substantial relationship (green cells); 0.70 - 0.90 - high correlation; marked relationship (brown cells); 0.90 - 1.00 - very high correlation; very dependable relationship (black cells).

Table S3. Correlation values according to Guilford [69] between the transcription level of the evaluate genes related to different processes under additional far-red light.

	Table S3	Photoreceptors				Circadian clock			Redox-responsive TFs			Nitrate reduction					Glutathione metabolism				Antioxidant enzymes						Thiols					
		PhyA	Cry1a	Cry1b	Cry2	CCA1	TOC1	PRR73	OXS2	ARF-like	mTREF	NR	GLNa	GLNb	GLNd	GLNe	APSR	γ-ECS	GST	GR	APX1	APX4	CAT1	CAT2	CAT4	PRX	CyS	CySS	$E_{CyS/CySS}$	GSH	GSSG	$E_{GSH/GSSG}$
Photoreceptors	PhyA		0.21	0.26	-0.34	-0.15	-0.02	-0.15	0.03	0.20	-0.18	0.02	-0.22	0.05	-0.06	-0.09	-0.41	-0.04	-0.14	-0.04	0.10	-0.10	-0.13	0.01	-0.14	-0.20	0.47	-0.10	-0.37	0.16	-0.26	-0.31
	Cry1a			0.92	0.04	0.47	-0.08	0.06	0.39	-0.08	0.63	0.44	0.24	0.43	0.25	-0.02	0.26	0.19	0.20	0.16	0.43	0.30	-0.10	0.24	0.42	0.16	0.21	-0.48	-0.40	-0.10	-0.28	-0.09
	Cry1b				-0.11	0.49	-0.21	0.02	0.45	0.04	0.64	0.50	0.23	0.47	0.23	-0.18	0.16	0.19	0.01	0.13	0.51	0.31	-0.07	0.29	0.47	0.09	0.13	-0.45	-0.45	0.05	-0.47	-0.30
	Cry2					-0.24	0.04	0.60	-0.31	-0.13	0.06	-0.41	0.16	-0.33	-0.19	0.29	0.06	-0.09	0.72	-0.03	-0.33	-0.14	0.28	-0.23	-0.22	-0.16	0.11	0.00	0.00	0.02	0.25	0.20
Circadian clock	CCA1						-0.29	-0.15	0.42	-0.22	0.36	0.48	0.25	0.44	0.28	-0.24	0.63	0.13	-0.13	0.03	0.37	0.38	-0.25	0.19	0.52	0.31	-0.14	-0.23	-0.13	-0.21	-0.25	-0.05
	TOC1							-0.09	0.52	0.44	-0.08	0.27	0.30	0.52	0.68	0.37	0.19	0.76	-0.06	0.74	0.53	0.54	0.16	0.66	0.28	0.41	-0.05	-0.01	-0.06	-0.07	0.17	0.14

	**PRR73**								-0.14	0.10	0.13	-0.24	0.48	-0.10	-0.03	0.41	0.05	0.11	0.60	0.17	-0.15	0.12	0.57	-0.02	0.02	-0.03	0.12	-0.18	-0.15	0.31	-0.12	-0.18
**Redox-responsive TFs**	**OXS2**									0.50	0.31	0.82	0.64	0.98	0.88	0.04	0.47	0.87	-0.30	0.81	0.94	0.87	0.17	0.92	0.87	0.67	-0.16	-0.23	-0.33	-0.01	-0.42	-0.25
	**ARF-like**										-0.15	0.17	0.24	0.49	0.33	0.23	-0.11	0.52	-0.21	0.53	0.45	0.33	0.53	0.53	0.17	0.19	-0.18	0.22	-0.16	0.27	-0.42	-0.44
	**mTREF**											0.41	0.31	0.30	0.23	0.01	0.28	0.16	-0.16	0.10	0.39	0.29	-0.08	0.21	0.45	0.17	-0.13	-0.43	-0.20	0.17	-0.20	-0.20
**Nitrate reduction**	**NR**												0.51	0.86		-0.05	0.34	0.67	-0.37	0.51	0.86	0.80	-0.15	0.81	0.84	0.56	-0.01	-0.40	-0.34	0.08	-0.47	-0.29
	**GLNa**													0.62	0.66	0.26	0.41	0.70	0.22	0.71	0.52	0.77	0.45	0.64	0.78	0.68	-0.02	-0.37	-0.31	0.10	-0.30	-0.16
	**GLNb**														0.91	0.12	0.44	0.88	-0.25	0.77	0.95	0.90	0.14	0.94	0.83	0.62	-0.07	-0.30	-0.38	0.01	-0.43	-0.26
	**GLNd**															0.18	0.45	0.95	-0.16	0.81	0.90	0.95	0.08	0.96	0.80	0.59	-0.02	-0.34	-0.35	0.03	-0.26	-0.13
	**GLNe**																-0.11	0.24	0.26	0.24	0.05	0.16	0.49	0.19	-0.06	0.17	0.09	-0.05	0.05	-0.04	0.22	0.17

Glutathione metabolism	**APSR**																0.40	0.10	0.50	0.33	0.46	0.10	0.33	0.59	0.54	-0.09	-0.35	-0.20	-0.02	-0.15	-0.03
	**γ-ECS**																	-0.08	0.91	0.87	0.93	0.28	0.96	0.74	0.56	-0.06	-0.24	-0.35	0.09	-0.26	-0.18
	**GST**																		0.02	-0.36	-0.09	0.26	-0.25	-0.17	-0.09	0.37	-0.18	-0.14	-0.17	0.28	0.34
	**GR**																			0.71	0.78	0.50	0.84	0.72	0.67	0.01	-0.27	-0.34	0.15	-0.27	-0.19
Antioxidant enzymes	**APX1**																				0.89	0.04	0.94	0.79	0.48	-0.10	-0.26	-0.36	0.10	-0.42	-0.30
	**APX4**																					0.12	0.93	0.85	0.58	-0.08	-0.33	-0.37	0.06	-0.31	-0.18
	**CAT1**																						0.21	0.13	0.23	0.11	-0.11	-0.19	0.36	-0.29	-0.31
	**CAT2**																							0.80	0.57	-0.06	-0.26	-0.36	0.14	-0.39	-0.28
	**CAT4**																								0.74	-0.10	-0.42	-0.35	0.08	-0.44	-0.25

	**PRX**																										-0.20	-0.17	-0.02	-0.17	-0.08	0.09
Thiols	**CyS**																											-0.63	-0.67	0.36	-0.21	-0.25
	**CySS**																												0.80	-0.30	0.36	0.28
	**$E_{CyS/CySS}$**																													-0.45	0.54	0.50
	**GSH**																														-0.68	-0.87
	**GSSG**																															0.93
	**$E_{GSH/GSSG}$**																															

* If correlation value is: < 0.20 - slight; almost negligible relationship; 0.20 - 0.40 - low correlation; definite but small relationship; 0.40 - 0.70 - moderate correlation; substantial relationship (green cells); 0.70 - 0.90 - high correlation; marked relationship (brown cells); 0.90 - 1.00 - very high correlation; very dependable relationship (black cells).

Table S4. Primer sequences of the target genes and their origin used for qPCR analysis.

Target Gene	Gene ID (NCBI nr)	Ensembl Plants ID	Strand	Primer Sequences (5' → 3')
*HvCyclophilin**	AK253120.1	HORVU6Hr1G012570	Forward	CCTGTCGTGTCGTCGGTCTAAA
			Reverse	ACGCAGATCCAGCAGCCTAAAG
HvPHYA	DQ201139.1 DQ201140.1, GU994113.1, GU994114.1	HORVU4Hr1G008610	Forward	TGCAGCACATTCAGAGAGGG
			Reverse	CCCTAGTGCCTTGTGCAGAG
HvCRY1a	DQ201149.1 DQ201150.1 DQ201151.1	not available	Forward	CACATGGAAGTGGACCGTGC
			Reverse	CGCTGGCAACTTGTTCCCTG
HvCRY1b	DQ201152.1 DQ201153.1 DQ201154.1	not available	Forward	GATGGAAGTCCATTGGGAACC
			Reverse	CATACGGTGCTGAGGTTGCTG
HvCRY2	DQ201155.1 DQ201156.1	HORVU6Hr1G058740	Forward	GGCTCGCGAGTGCTTAGATG
			Reverse	GGAGCACTTGGAGATAGTTCCTTC
HvCCA1	JN603242.1	HORVU7Hr1G070870	Forward	AATAAGACTGGGGCAACTGGC
			Reverse	TAGTTGTGGGGAAAGGGCTG
HvTOC1	JN603243.1	HORVU6Hr1G057630	Forward	AGGCAGAAAAGGAAGGACCG
			Reverse	TGCCTAACAAACTGACCCCG
HvPRR73	AK356430.1	HORVU5Hr1G081620	Forward	GGTGAAGGGTCAGTTTGTT
			Reverse	ACGACAAGTTAGCGTATACAA
HvOXS2	AK375304.1	HORVU4Hr1G005770	Forward	TCTGGGTCGCCAATGAACTC
			Reverse	GCTCCTCATTGTCTGCTGGT
HvARF-like	AK358522.1	HORVU4Hr1G011740	Forward	GATGGGTGTATGTGTGTACTT
			Reverse	CTTGCTCTGAGGCTGTTATT
HvmTREF	AK360739.1	HORVU5Hr1G103380	Forward	CGACCATGGTTTGTGATGTA
			Reverse	CCTATACACATGGCACATCC
HvNR	AK365506.1		Forward	ACGTGGAGCTGCTCATCAAG

		HORVU6Hr1G079700	Reverse	TCGAGGTACTGCGACATGAG
HvGLN_a	AK252215.1	HORVU1Hr1G038060	Forward	TTTGATGGGTGCGCAAATCC
			Reverse	GGTGAATGAGATCGCCAAATGC
HvGLN_b	AK365634.1	HORVU2Hr1G111300	Forward	ATTTGCGCACATTGCTCTGC
			Reverse	CAGCGCATCCTTCAAGTTGTTG
HvGLN_d	JX878490.1	HORVU4Hr1G066860	Forward	TCGACATCAGGAGCAAAGCAAG
			Reverse	CTGAACGGGTCCTTGAAAATGG
HvGLN_e	KF815944.1	HORVU6Hr1G074030	Forward	TATTTTGCTGACGGCGAACG
			Reverse	ATTCAGGAGCAAGAAGGCGAAG
HvAPSR	not available	not available	Forward	TCGCCTCTGAGATCATGGA
			Reverse	GTCCAGTCAGCTTCGCGTAT
Hvγ-ECS	AK353769.1	HORVU1Hr1G015590	Forward	TGCCCACTTTGAACGATTGG
			Reverse	TCCCGGAATGGTGTCTTCAAAC
HvGST	AK251838.1	HORVU5Hr1G103430	Forward	CGGAAGGAAGCTACAGATAAA
			Reverse	GACACATGACACACACATAGA
HvGR	AK376974.1	HORVU6Hr1G089780	Forward	TGGAGGCTACTTGCTTTGCT
			Reverse	AGATAGCGGCGGAATACAGA
HvAPX1	MF804856.1	HORVU7Hr1G057430	Forward	CAGTGGCCCAGCATCTACAA
			Reverse	TAGCCCGCGATAACTACAGC
HvAPX4	AK356559.1	HORVU2Hr1G101730	Forward	AGGCCCCATTTCATTTGCAG
			Reverse	CGCCCGAATATTTTGTCGAAG
HvCAT1	AF021939.1	HORVU6Hr1G008640	Forward	AAGTGCGGCTTCAAGAACAACC

			Reverse	TCGTCTTCTCCCTCTTTCCAACC
HvCAT2	AK366177.1	HORVU7Hr1G121700	Forward	CGCGGAAAATGAACAGCTTG
			Reverse	GAGCACATTTCGGGGCATTC
HvCAT4	AK357533.1	HORVU4Hr1G082040	Forward	CAACCGCAACATCGACAACTTC
			Reverse	TTGTTGTGGTGGGAGCACTTG
HvPRX1	AK248281.1	HORVU3Hr1G077580	Forward	AGGCAACAAGAAGGACGAAACG
			Reverse	ACGTACGCGCTGCAGATTTTC

* kindly provided by Morran *et al.* [78].

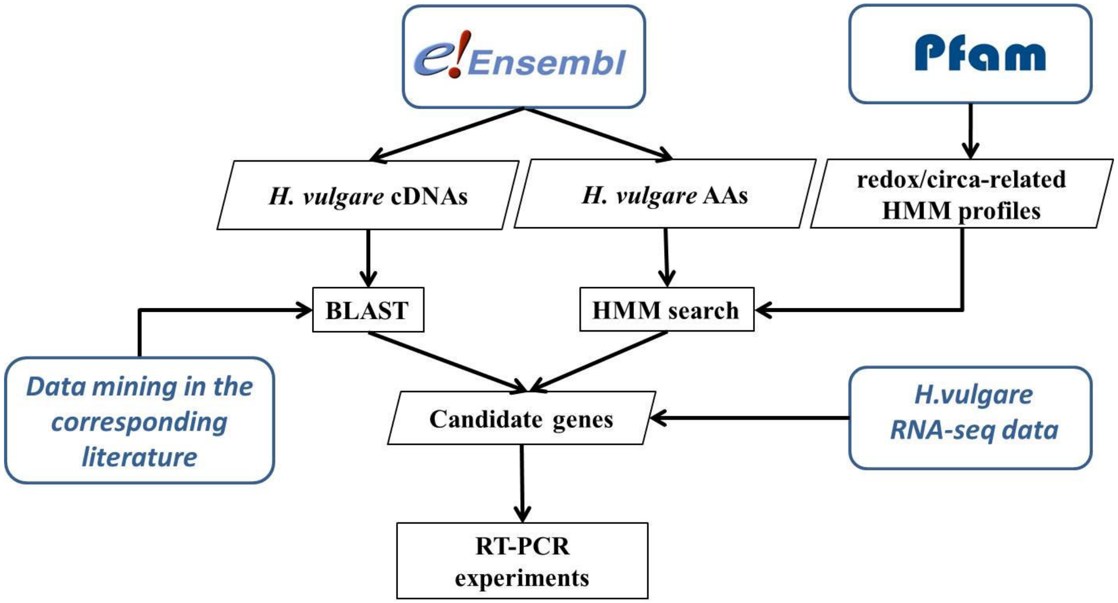

Figure S1. Selection procedure of redox-, circadian rhythm- and far-red-dependent genes. The *Hordeum vulgare* reference proteome was retrieved from Ensembl Plants FTP server (ftp.ensemblgenomes.org; release-41). The whole protein collection was subsequently scanned with the Hidden Markov Model (HMM)-based HMMER 3.0 software package http://eddylab.org/software/hmmer/) [74] using redox and circadian-related HMM profiles of the Pfam 32.0 database (ftp://ftp.ebi.ac.uk) [75]. We also performed selection of genes responsive to far-red light with high expression changes (Galiba and Cattivelli, unpublished RNA-seq data).

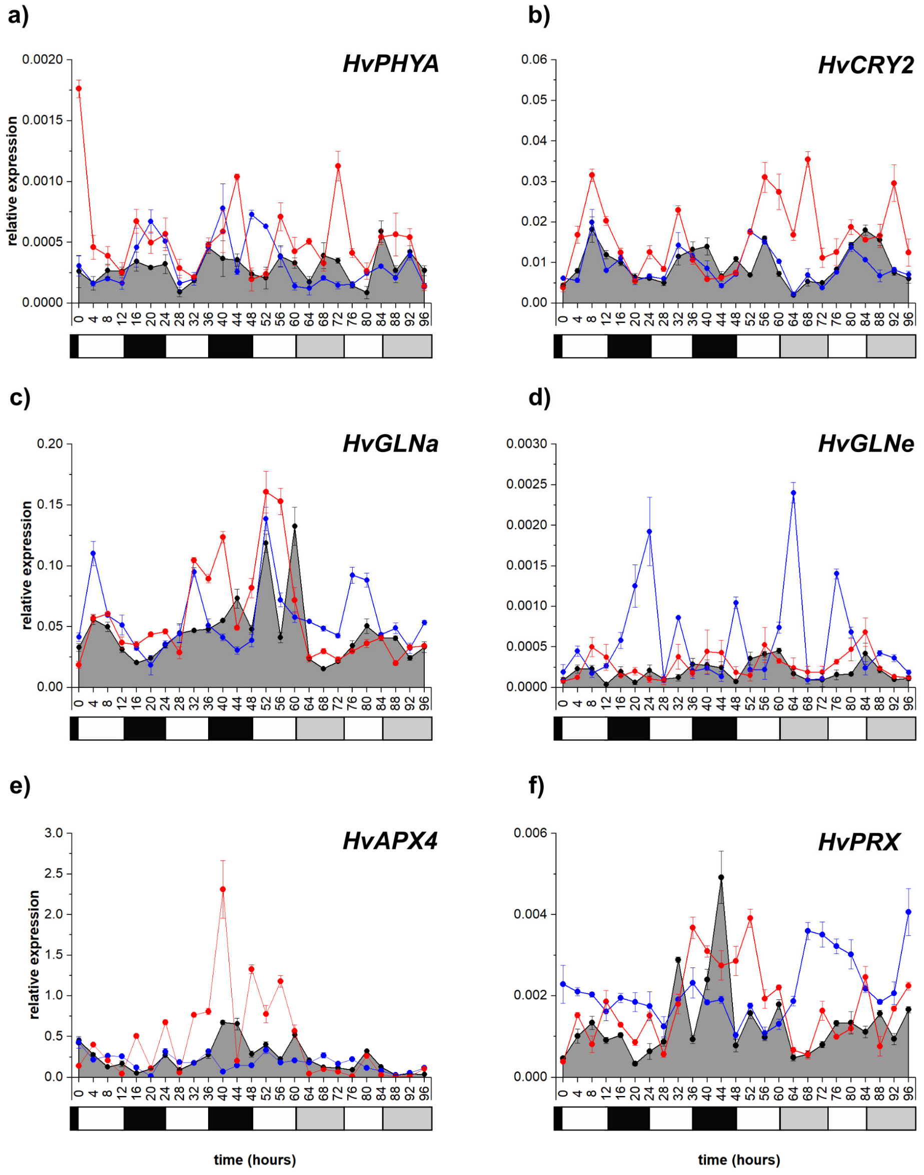
a)
HvPHYA
relative expression
b)
HvCRY2
c)
HvGLNa
relative expression
d)
HvGLNe
e)
HvAPX4
relative expression
f)
HvPRX
time (hours)
time (hours)

Figure S2. Expression patterns of *H. vulgare* phytochrome A (*HvPHYA*) (**a**), cryptochrome 2 (*HvCRY2*) (**b**), glutamine synthetase-a (*HvGLNa*) (**c**), glutamine synthetase-e (*HvGLNe*) (**d**), ascorbate peroxidase 4 (*HvAPX4*) (**e**) and peroxiredoxin (*HvPRX*) (**f**) coding genes under white, blue and far-red light illumination. Transcript levels were calculated with ΔCt method, expression data and error bars indicating standard deviations were calculated using three biological replicates. The following significant differences were calculated at $p \leq 5\%$ level: *HvPHYA* — 0.00012; *HvCRY2* — 0.0035; *HvGLNa* — 0.013; *HvGLNe* — 0.00019; *HvAPX4* — 0.21; *HvPRX* — 0.00016.

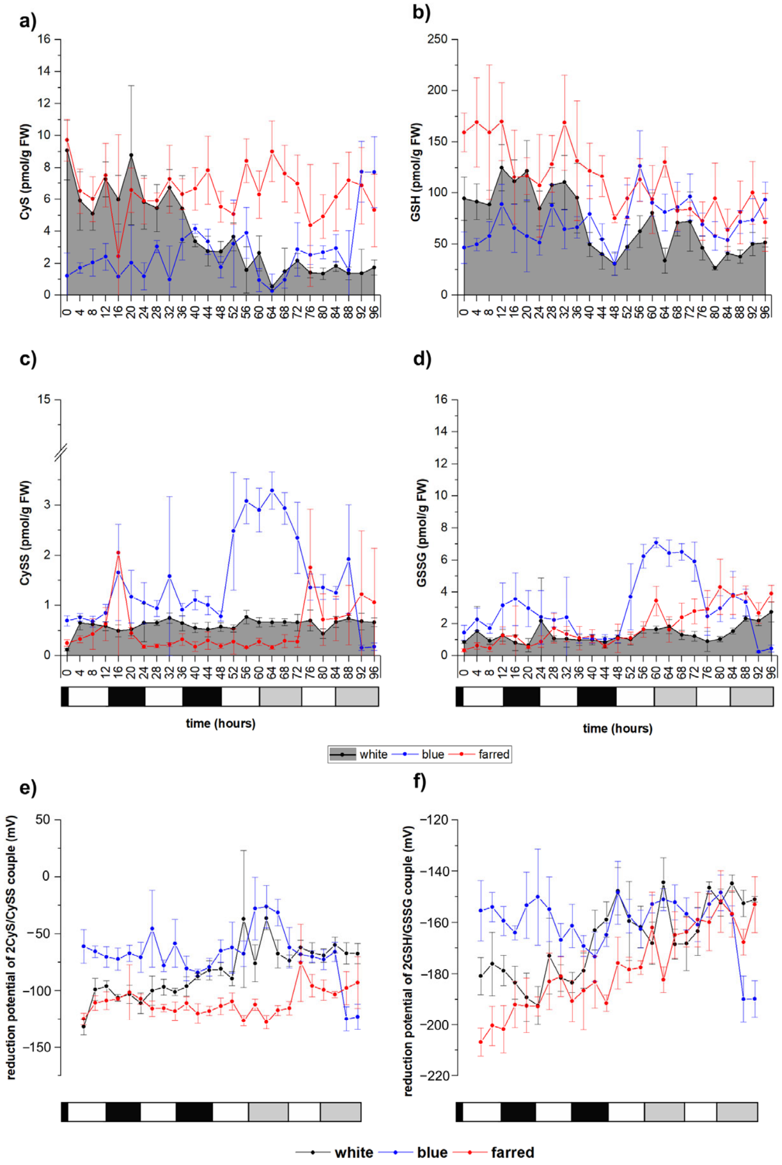
a)
b)
c)
d)
e)
f)
CyS (pmol/g FW)
GSH (pmol/g FW)
CySS (pmol/g FW)
GSSG (pmol/g FW)
reduction potential of 2CyS/CySS couple (mV)
reduction potential of 2GSH/GSSG couple (mV)
time (hours)
white
blue
farred

Figure S3. Changes in cysteine (**a**), glutathione (**b**), oxidized cysteine (**c**) and glutathione disulfide (**d**) content and reduction potential (E) of Cys/CySS (**e**) and GSH/GSSG (**f**) redox-couples in response to additional B/FR light treatment. Values on the X-axes show the time in hours after the start of the experiment; white and back bars below them indicate the light and dark periods, while grey bars indicate the subjective "night" period during constant light conditions. Data and error bars indicating standard deviations were calculated using three biological replicates. The following significant differences were calculated at $p \leq 5\%$ level: Cys: — 0.56; GSH: — 12.4; CySS — 0.16; GSSG — 0.23; $E_{2Cys/CySS}$ — 24,2; $E_{2GSH/GSSG}$ — 21,9.
